# Comprehensive immunoproteogenomic analyses of malignant pleural mesothelioma

**DOI:** 10.1172/jci.insight.98575

**Published:** 2018-04-05

**Authors:** Hyun-Sung Lee, Hee-Jin Jang, Jong Min Choi, Jun Zhang, Veronica Lenge de Rosen, Thomas M. Wheeler, Ju-Seog Lee, Thuydung Tu, Peter T. Jindra, Ronald H. Kerman, Sung Yun Jung, Farrah Kheradmand, David J. Sugarbaker, Bryan M. Burt

**Affiliations:** 1 Division of Thoracic Surgery, Michael E. DeBakey Department of Surgery,; 2 Department of Biochemistry and Molecular Biology,; 3 Section of Hematology-Oncology, Department of Medicine,; 4 Department of Radiology, and; 5 Department of Pathology and Immunology, Baylor College of Medicine, Houston, Texas, USA.; 6 Department of Systems Biology, Division of Cancer Medicine, The University of Texas MD Anderson Cancer Center, Houston, Texas, USA.; 7 Division of Abdominal Transplantation, Immune Evaluation Laboratory, Michael E. DeBakey Department of Surgery, and; 8 Department of Medicine, Baylor College of Medicine, Houston, Texas, USA.; 9 Center for Translational Research in Inflammatory Diseases, Michael E. DeBakey VA Medical Center, Houston, Texas, USA.

**Keywords:** Immunology, Oncology, Cancer immunotherapy, Expression profiling, Proteomics

## Abstract

We generated a comprehensive atlas of the immunologic cellular networks within human malignant pleural mesothelioma (MPM) using mass cytometry. Data-driven analyses of these high-resolution single-cell data identified 2 distinct immunologic subtypes of MPM with vastly different cellular composition, activation states, and immunologic function; mass spectrometry demonstrated differential abundance of MHC-I and -II neopeptides directly identified between these subtypes. The clinical relevance of this immunologic subtyping was investigated with a discriminatory molecular signature derived through comparison of the proteomes and transcriptomes of these 2 immunologic MPM subtypes. This molecular signature, representative of a favorable intratumoral cell network, was independently associated with improved survival in MPM and predicted response to immune checkpoint inhibitors in patients with MPM and melanoma. These data additionally suggest a potentially novel mechanism of response to checkpoint blockade: requirement for high measured abundance of neopeptides in the presence of high expression of MHC proteins specific for these neopeptides.

## Abstract 2

A systematic immunoproteogenomic investigation of malignant pleural mesothelioma reveals mechanisms of anti-tumor immunity.

## Introduction

Malignant pleural mesothelioma (MPM) is a highly aggressive malignancy of the pleura that is fatal in most cases and has defied standard approaches to treatment. Palliative therapy is associated with median survival of 7 months (1), while cytotoxic systemic therapy combined with targeted therapy (bevacizumab) can extend survival to 18 months (2). In selected patients with early-stage disease, surgery-based multimodal approaches incorporating cytotoxic chemotherapy and/or radiotherapy may improve median survival to 26 months (3). In early-phase clinical trials, immune checkpoint inhibitors targeting programmed cell death 1 (PD-1) have recently shown encouraging clinical activity with good tolerability in patients with advanced MPM who progressed after first-line chemotherapy (4–6). Similar to the results of checkpoint blockade in other human tumors (7, 8), however, less than 50% of patients with MPM will benefit from PD-1 inhibition.

The variance in clinical responses to checkpoint inhibition emphasizes the complexities of productive antitumor immunity. Whereas we and others have shown that individual immune cell types influence the clinical behavior of MPM (9, 10), these cells have been studied in relative isolation, outside of their complex cellular communities. The high-dimensional, single-cell platform time-of-flight mass cytometry (CyTOF) has substantially augmented the ability of single-cell cytometry to investigate the complex cellular networks of human disease states (11–13). By utilizing probes conjugated to stable heavy metal isotopes, CyTOF avoids the signal overlap inherent to the fluorophore-conjugated antibodies of conventional flow cytometry and enables simultaneous quantification of over 40 parameters at single-cell resolution with minimal signal overlap. Further, data-driven analysis techniques have facilitated straightforward analyses and graphical representation of these complex data (14, 15). Thus, it is now possible to discern the identity and behavior of numerous cell types from a single experiment and to contextualize these individual metrics into a broader immunologic state. These features have substantially augmented the ability of single-cell cytometry to investigate the complex cellular systems and processes that influence tumor biology (11–13) and uniquely position CyTOF to identify the cellular networks that are responsible for successful immunotherapy. We hypothesized that dissection of the intratumoral cellular networks within MPM would define distinct immunologic subtypes of this tumor and would generate a framework for investigation of the immunogenomic mechanisms responsible for cancer-related patient outcomes and response to PD-1 inhibition.

## Results

### Mass cytometry identifies 2 distinct immune microenvironments in MPM.

**Figure 1:**
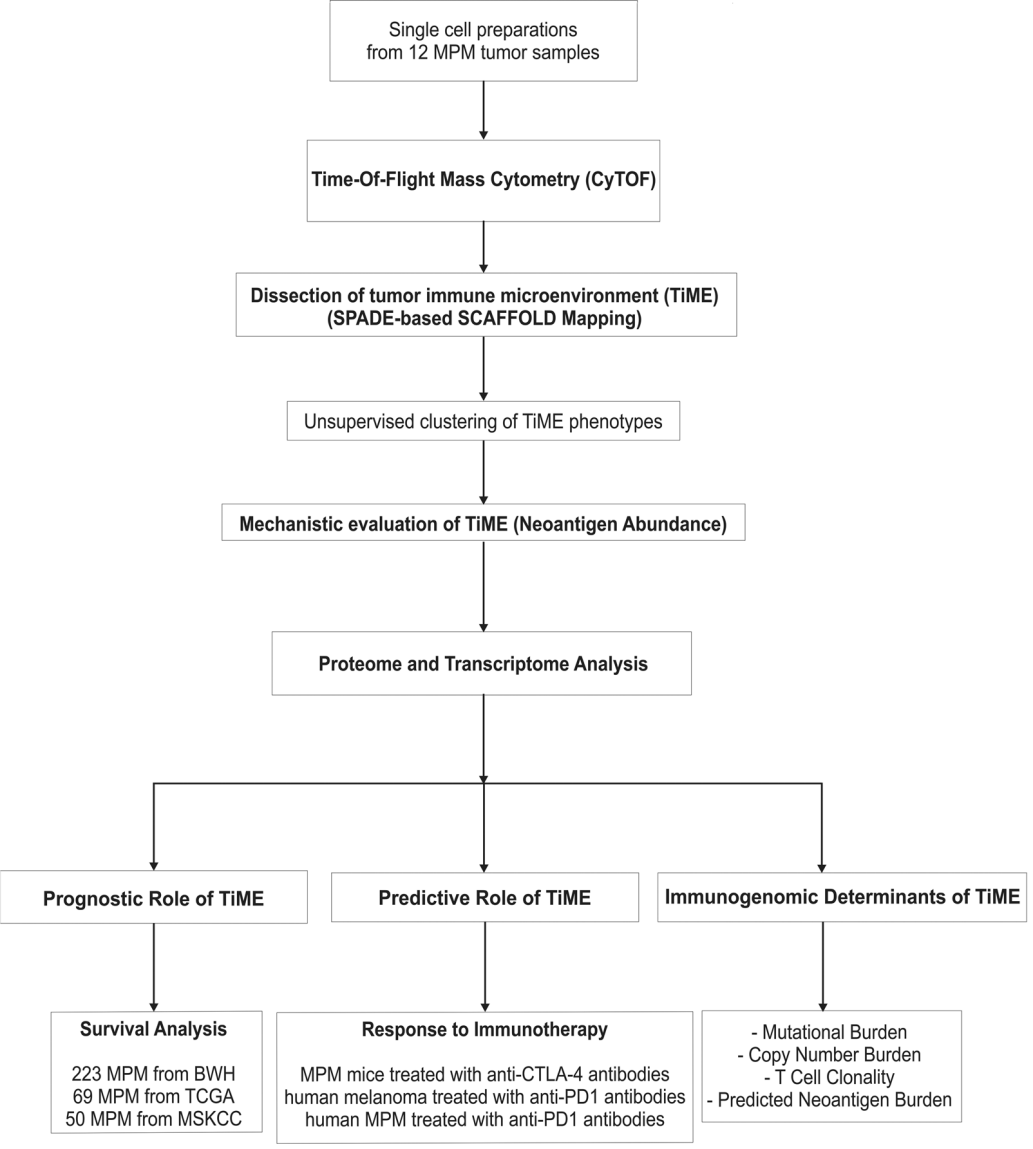
Schematic illustration of study design. BWH, Brigham and Women’s Hospital; CTLA-4, cytotoxic t-lymphocyte associated protein 4; CyTOF, time-of-flight mass cytometry; MPM, malignant pleural mesothelioma; MSKCC, Memorial Sloan Kettering Cancer Center; PD-1, programmed cell death 1; SCAFFOLD, single-cell analysis by fixed force– and landmark-directed; SPADE, spanning-tree progression analysis of density-normalized events; TCGA, The Cancer Genome Atlas; and TiME, tumor immune microenvironment.

**Figure 2:**
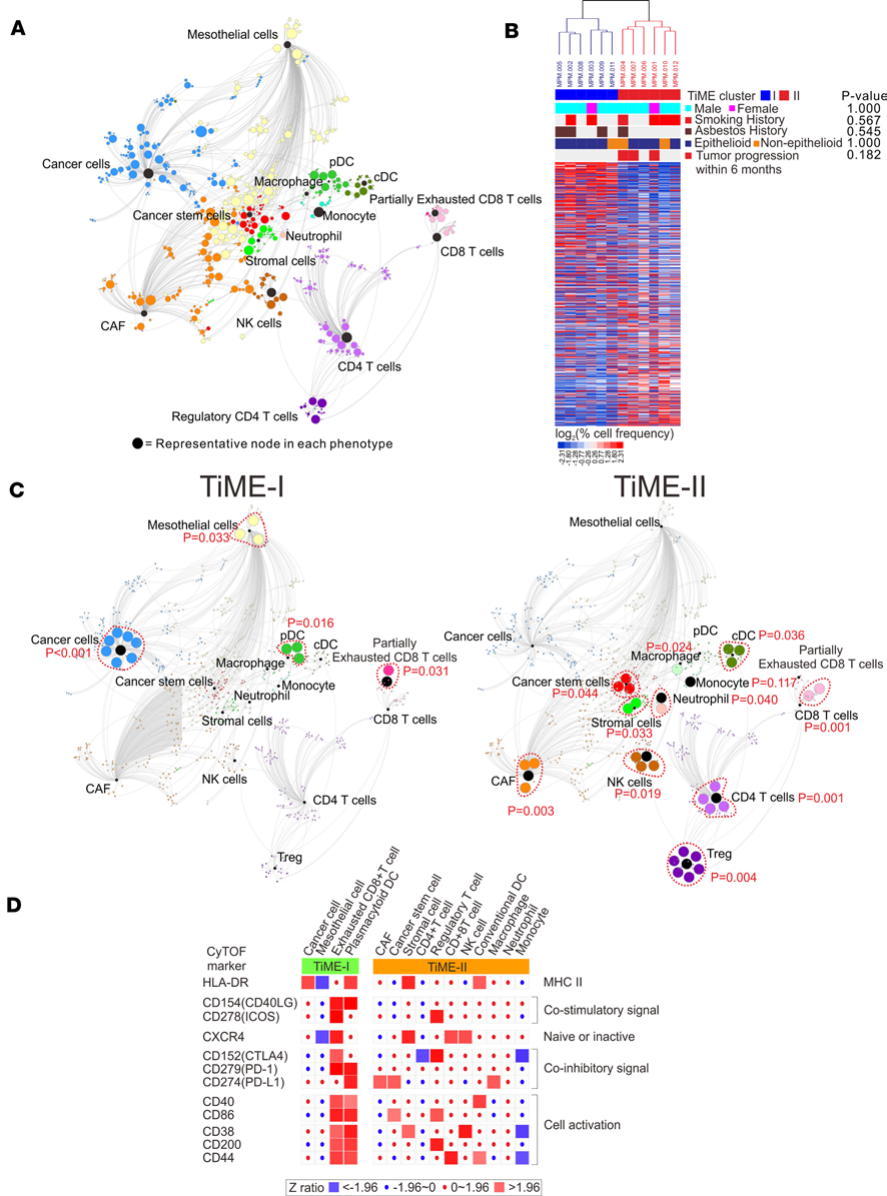
SCAFFOLD maps of tumor immune microenvironment (TiME) in MPM. (**A**) A SCAFFOLD map of TiME in 12 human MPM tumors. CyTOF was performed on 12 MPM tumors utilizing a panel of 35 metal-conjugated antibodies. Pooled data from these 12 patients was used to generate a SCAFFOLD reference map of MPM’s intratumoral immune system. This approach provides a data-driven representation of cellular networks, while also denoting the location of landmark immune cell populations defined using prior knowledge of the immune system. For example, landmark nodes are visualized as black nodes and represent 15 manually defined major cellular phenotypes. The same cells are subjected to unsupervised clustering to provide an objective view of cell composition and organization, and 742 cellular subpopulations were identified and represented by the colored nodes. In these maps, node size represents the relative number of cells in that grouping, and line length indicates similarity between cells. In other words, 2 groups of cells are connected by a short line if the proteins they express are relatively similar, and a longer line if they are relatively disparate. (**B**) Two distinct subsets of MPM patients were identified by unsupervised clustering of pooled CyTOF data from 12 MPM tumors: 6 tumors of the TiME-I subset and 6 tumors of the TiME-II subset. (**C**) The SCAFFOLD maps of TiME-I and TiME-II subsets. SCAFFOLD maps were generated from pooled data from the 6 patients in each of the TiME-I and -II immunologic subsets of MPM, and cellular subpopulations were statistically compared between each subset. The internodal differences in the same phenotypes were analyzed with 2-tailed paired *t* test according to the corresponding nodes. (**D**) Differential activation states of the immune cell populations between TiME-I and TiME-II MPM tumors. Immune stimulatory or inhibitory markers were significantly altered between 2 TiME subsets. Z ratios were calculated by taking the difference between the averages of the observed marker Z scores and dividing by the SD of all the differences for that particular comparison. A Z ratio of ±1.96 was inferred as significant (*P* < 0.05). CAF, cancer-associated fibroblast; cDC, conventional DCs; CyTOF, time-of-flight mass cytometry; MPM, malignant pleural mesothelioma; pDC, plasmacytoid DCs; SCAFFOLD, single-cell analysis by fixed force– and landmark-directed; TiME, tumor immune microenvironment; and Treg, CD4^+^ Tregs.

Tumor tissues from 12 consecutive treatment-naive MPM patients undergoing surgical resection were prepared for CyTOF, mass spectrometry (MS), and mRNA transcriptome profiling. The study scheme and patient characteristics are illustrated in Figure 1 and Supplemental Table 1 (supplemental material available online with this article; https://doi.org/10.1172/jci.insight.98575DS1). The tumor immune microenvironment (TiME) of MPM was characterized using CyTOF with a 35-antibody panel (Supplemental Table 2) and a single-cell analysis by fixed force– and landmark-directed (SCAFFOLD) map (16) was generated from 742 cellular subpopulations (nodes) based on 15 cellular phenotypes (Figure 2A, Supplemental Figures 1–4). Unsupervised clustering of these cellular subpopulations (17) defined 2 vastly disparate immunologic subtypes of MPM tumors, referred to as TiME-I and TiME-II subtypes (*n* = 6 in each subset) (Figure 2B). We next generated SCAFFOLD maps for TiME-I and -II MPM to compare differences in the frequency of major cellular subpopulations between these subtypes (Figure 2C), and a heatmap was generated to further distinguish these subtypes by differential activation states of their immune cell populations (Figure 2D). The TiME-I subtype contained significantly greater numbers of partially exhausted CD8^+^ T cells (PD-1^+^CTLA-4^+^CD8^+^ T cells), which have been shown to respond to checkpoint blockade through the release of IFN-γ (18, 19). Consistent with these reports, we found that PD-1^+^CTLA-4^+^CD8^+^ T cells in MPM had the ability to produce higher levels of IFN-γ and demonstrated increased phosphorylation of transcription factors including ERK, p38, and STAT4, when compared with non–PD-1^+^CTLA-4^+^CD8^+^ T cells (Supplemental Figures 5 and 6). TiME-I tumors also contained a greater frequency of HLA-DR^+^ cancer cells, which positively correlate with response to checkpoint blockade in melanoma patients (20) and which we have found to demonstrate increased expression of cytokines (IL-10, IL-6, and TNF-α) and phosphorylated transcription factors (HIF-1A, cPARP, and STAT3) in MPM (Supplemental Figure 5). We also identified plasmacytoid DC (pDC) in the TiME-I subtype expressing high levels of CD40 and CD86 (Figure 2, C and D). In contrast, TiME-II tumors contained more Tregs expressing high ICOS and CTLA-4 markers, as well as CXCR4^+^CD38^–^ (naive) CD8^+^ T cells (21). Also increased in TiME-II tumors were neutrophils, conventional DCs (cDC), cancer-associated fibroblasts (CAF), and tumor-associated macrophages (TAM) with high PD-L1, which were associated with greater IL-10 production and phosphorylation of Akt and NF-κβ (Figure 2D and Supplemental Figure 5).

### Neoantigen abundance and MHC protein expression underlie TiME.

**Figure 3:**
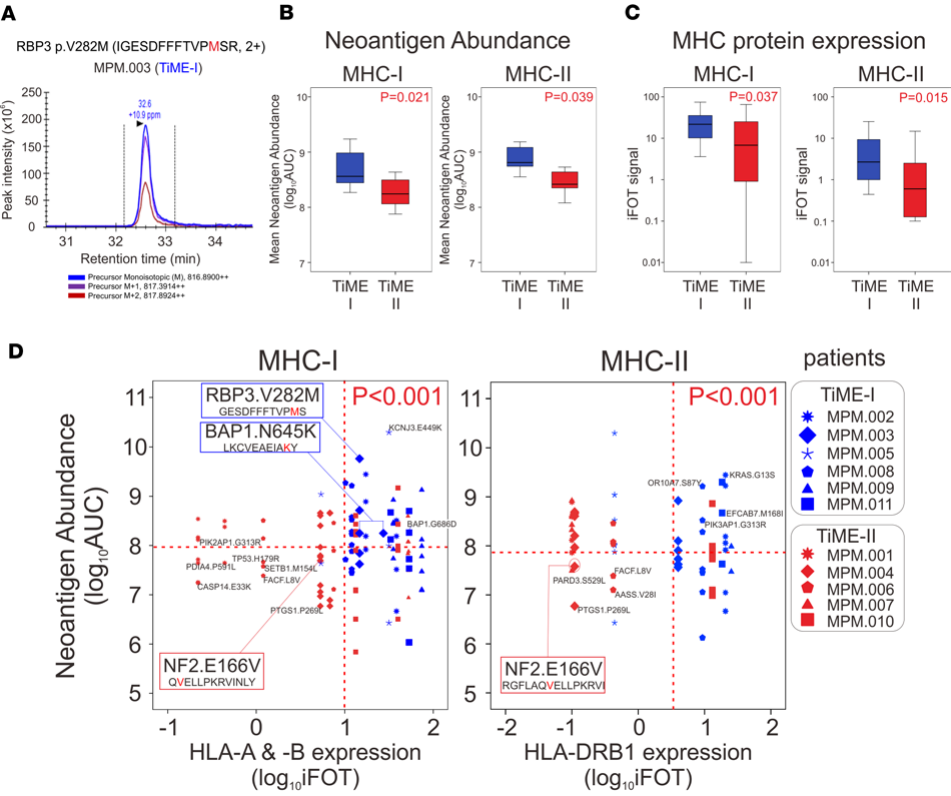
Neoantigen abundance and corresponding MHC molecules between 2 distinct TiME subsets. (**A**) Direct identification of neoantigen abundance of IGESDFFFTVP**M**SR of RBP3^V282M^ by mass spectrometry. Triple-redundant peaks of monoisotopic ^12^C, ^13^C (M+1), and ^14^C (M+2) support that the identified peaks for the peptides are accurately made. (**B**) Mean neoantigen abundance of directly identified neopeptides for MHC-I and MHC-II was determined by mass spectrometry on 11 MPM tumors (*n* = 6 TIME-I and *n* = 5 TiME-II). The 2-tailed Student’s *t* tests were used to compare the data. (**C**) MHC-I and MHC-II protein expression was determined by mass spectrometry on 11 MPM tumors. The 2-tailed Student’s *t* tests were used to compare the data. (**D**) Two-dimensional plots between abundance of neopeptides with high affinity to HLA-A, HLA-B, and HLA-DRB1 and the expression of the specific corresponding MHC molecules, on 11 MPM tumors. The 2-tailed χ^2^ tests were used to compare the data. BAP1, BRCA1 associated protein 1; iFOT, fraction of total intensity based absolute quantification; MHC, major histocompatibility complex; MPM, malignant pleural mesothelioma; NF2, neurofibromin 2; RBP3, retinol binding protein 3; and TiME, tumor immune microenvironment.

Neoantigens have recently been shown to influence the clinical behavior and response to immunotherapy in a number of human malignancies (22–26). Determination of neoantigen burden, in this context, has relied exclusively on exome and transcriptome analyses and in silico prediction of the presence of neoantigens. MS has recently been applied to human tumors for direct identification of neoantigens (27), and we utilized MS to compare measured neoantigen loads between TiME-I and -II immunologic subsets of MPM. Whereas it may be ideal to perform whole exome sequencing (WES) to quantify tumor mutation burden on an individual patient basis, this methodology may not be feasible or expedient at the scale of clinical practice. Therefore, potentially novel approaches to measure tumor mutation burden have been investigated by others through targeted next-generation sequencing (28, 29) and by utilizing the Catalogue Of Somatic Mutations In Cancer (COSMIC; http://cancer.sanger.ac.uk/cosmic) mutation database (30–32). The correlation of tumor mutation burden determined by targeted sequencing with tumor mutation burden determined by WES provided rationale for our development of a mesothelioma-specific mutation database to apply clinically in the prediction of response to PD-1 blockade. From next-generation sequencing data from a total of 640 tumors in the Brigham Women’s Hospital (BWH) (33) and Memorial Sloan Kettering Cancer Center (MSKCC) (34) cohorts and the COSMIC database (http://cancer.sanger.ac.uk/cosmic) (35), we generated a reference database containing 2,299 missense mutation sites in 1,885 genes where a detected mutation alters the amino acid sequence (Supplemental Figure 7 and Supplemental Table 3) in order to create a list of potential mutated peptides. MS was then performed on 11 of the 12 MPM tumors, which underwent CyTOF (described above), and blood samples from these patients were utilized for molecular HLA typing. A total of 140 mutated peptides were detected among these 11 tumors by MS, to which we applied standard prediction algorithms of the Immune Epitope Database (IEDB; http://www.iedb.org/) (36) to identify potential MHC class I (37, 38) and class II neoantigens (39, 40). We found that the median number of potential neoantigens for MHC-I (19 neoantigens[range 9–27]) and MHC-II (16 neoantigens [range 9–23]) was not different between TiME-I and TiME-II tumors (each *P* > 0.05). AUC analyses of peak neoantigen intensities were performed to quantify each of the detected neoantigens, a metric that we termed neoantigen abundance (Supplemental Tables 4–7). As an example, the RBP3^V282M^ (GESDFFFTVPMS) neopeptide that is predicted to have high affinity to HLA-B*18:01 in patient MPM.003 (TiME-I) demonstrated high abundance in this tumor (Figure 3A). By calculating the average abundance of all neoantigens (average of all AUC values of neoantigens in each patient [sum AUCs divided by the number of neoantigens]), we were able to quantify a representative amount of measured neoantigens in each patient and study this metric as a predictive biomarker for response to checkpoint blockade. We then compared neoantigen abundance between TiME-I and -II MPM subtypes and found that the average neoantigen abundance for both MHC-I and MHC-II was greater in TiME-I tumors (Figure 3B). As neoantigen presentation to T cells requires MHC-I or -II proteins that may themselves have variable levels of expression, we utilized MS to additionally quantify the expression of total MHC-I (HLA-A, -B, and -C) and MHC-II proteins (HLA-DRB1, -DRB3, -DRB4, -DRB5, -DPA1, -DPB1, -DQA1, and -DQB1) in each tumor. These data demonstrated that TiME-I tumors had elevated levels of both MHC-I and -II proteins compared with TiME-II tumors (Figure 3C) and that the predominantly expressed MHC-I proteins were HLA-A and HLA-B and the predominant MHC-II protein was HLA-DRB1.

Because each neoantigen has specificity for distinct MHC proteins, we examined the relationship of each neoantigen’s abundance and the level of expression of its specific corresponding MHC protein. Compared with TiME-II tumors, TiME-I tumors contained more high-abundance neoantigens with concordant high expression of their specific MHC-I proteins and/or MHC-II proteins (Figure 3D, Supplemental Figures 8 and 9). For example, in a patient with a TiME-I tumor (MPM.003; HLA-A*01:01, HLA-A*30:02, and HLA-B*18:01), the BAP1^N645K^ neoantigen (LKCVEAEIA**K**Y) (derived from the most common gene mutation in MPM; ref. 33) was present in high abundance, and its corresponding MHC-I proteins were also present at high levels. In contrast, in a patient with a TiME-II tumor (MPM.004; HLA-A*26:01, HLA-DRB1*08:01, and HLA-DRB1*13:01), the NF2^E166V^ neoantigens (Q**V**ELLPKRVINLY and RGFLAQ**V**ELLPKRVI) with high predicted affinity to HLA-A and HLA-DRB1, respectively, were demonstrated at low abundance, along with low expression of its corresponding HLA proteins (Figure 3D).

### The TiME signature is a robust prognosticator in MPM.

**Figure 4:**
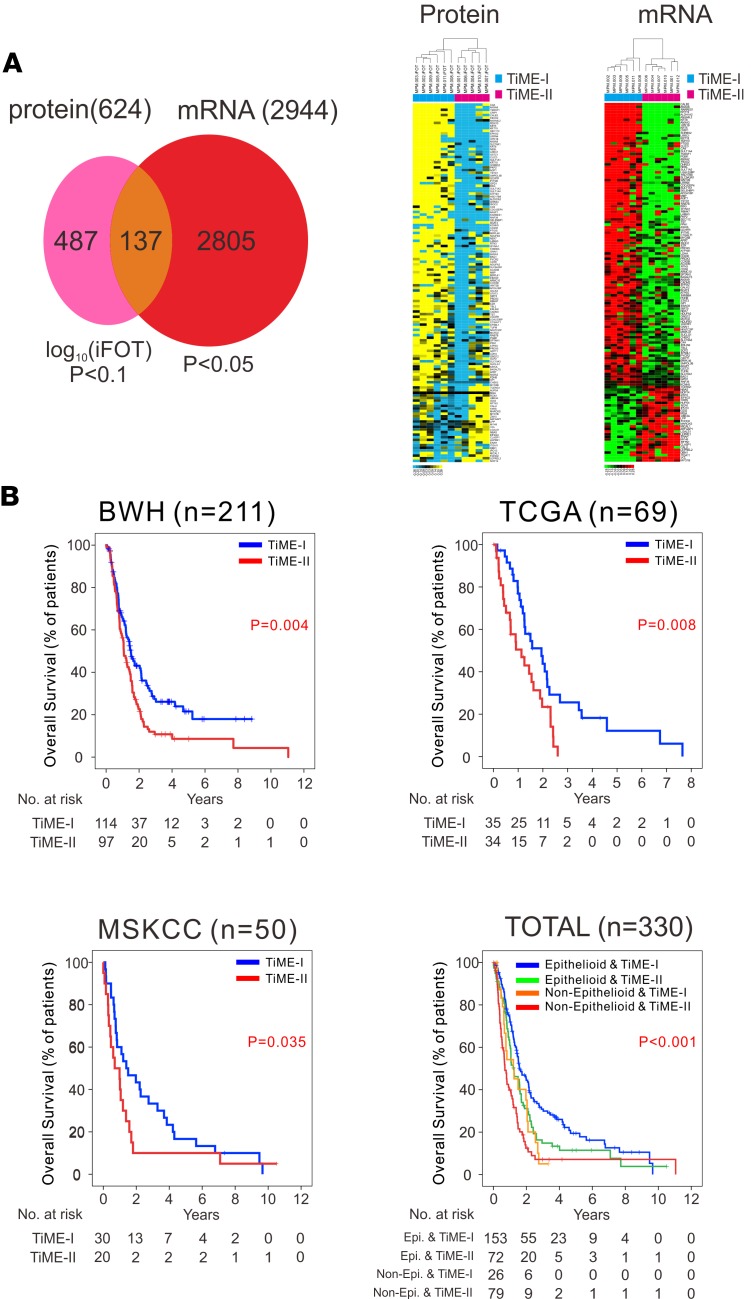
Prognostic significance of the TiME signature in MPM. (**A**) Development of a molecular signature that discriminates TiME-I and TiME-II MPM tumors through protein profiling by mass spectrometry and mRNA transcriptome analysis using 137 differential proteins, also differentially expressed in mRNA. (**B**) Kaplan-Meier curves of overall survival in the BWH cohort (*n* = 211), the TCGA MPM cohort (*n* = 69), the MSKCC cohort (*n* = 50), and a combined dataset (*n* = 330). Survival curves were generated with the Kaplan-Meier’s method, and intergroup comparisons were performed with the log-rank test. BWH, Brigham and Women’s Hospital; MPM, malignant pleural mesothelioma; MSKCC, Memorial Sloan Kettering Cancer Center; TCGA, The Cancer Genome Atlas; and TiME, tumor immune microenvironment.

Although a single-cell assay could be used as a platform to test the correlation of an immunologic signature with clinical outcomes (41), we reasoned that a molecular representation of TiME subsets in MPM may currently be a more practical and applicable approach. To achieve this goal, we performed mRNA microarrays and MS profiling on the same tumors that underwent CyTOF. Among 2,944 mRNAs with differential expression (*P* < 0.05) between the TiME-I and TiME-II subtypes, we selected 137 genes whose expression was also statistically different between the TiME subtypes at the protein level, which was defined as our molecular TiME signature (Figure 4A, Supplemental Figure 10, and Supplemental Table 8). We then evaluated the relationship of the TiME signature with prognosis in MPM by utilizing mRNA sequencing data of 211 MPM patients from the BWH cohort (33) and 69 MPM patients from The Cancer Genome Atlas (TCGA) portal (https://gdc-portal.nci.nih.gov/) (TCGA cohort), as well as mRNA microarray data of 50 patients from MSKCC cohort (34) (Supplemental Table 1). Significant association of the TiME signature with overall survival (OS) was demonstrated in each of these 3 independent MPM cohorts (Figure 4B), and multivariable analysis in a merged cohort (*n* = 330) accounting for age, asbestos exposure, histology (epithelial vs. nonepithelial), and pathologic stage revealed the TiME signature to be a robust independent prognostic factor in MPM (*P* = 0.017, hazard ratio = 1.74, 95% CI, 1.32–2.30) (Supplemental Table 9). Given the association of TiME-I and TiME-II subsets with favorable and unfavorable OS, respectively, the TiME-I subset is hereafter referred to as good-TiME and the TiME-II subset as bad-TiME.

### Good-TiME is associated with favorable responses to immune checkpoint blockade.

**Figure 5:**
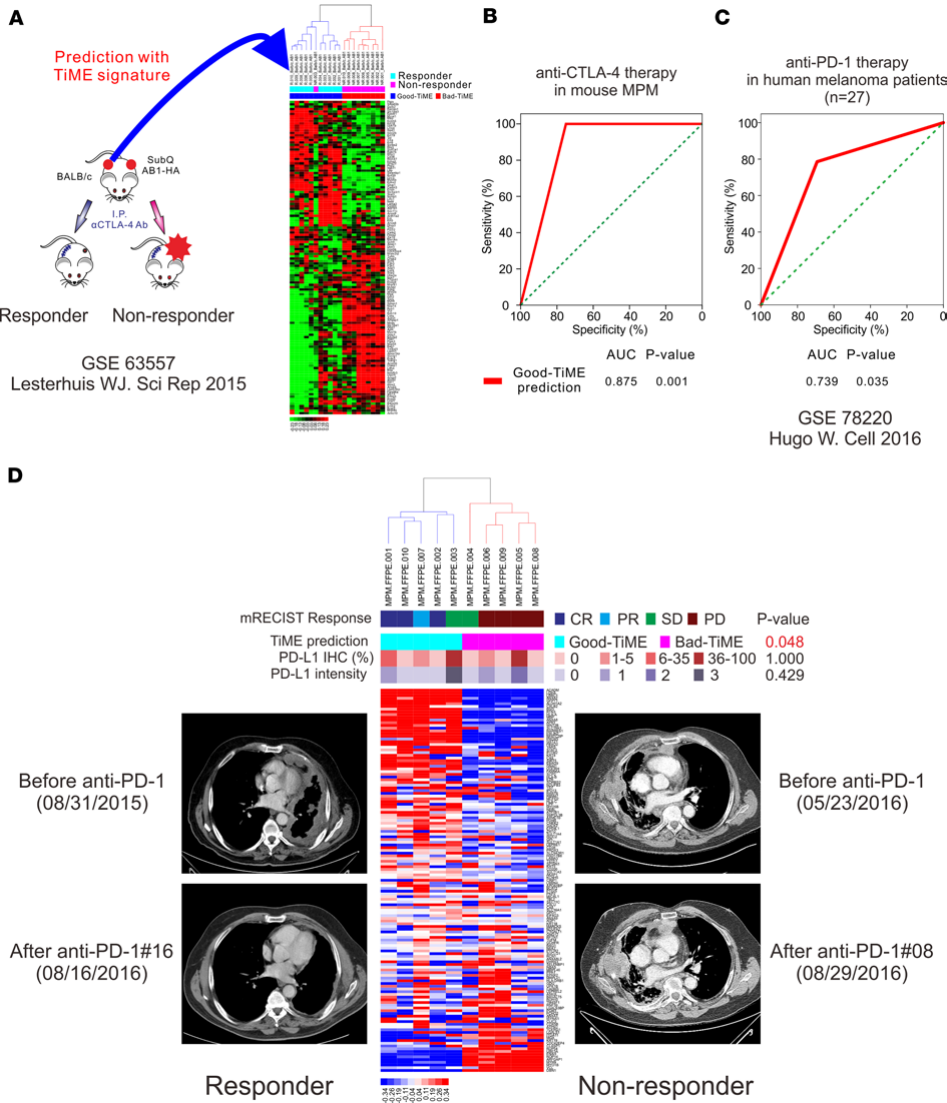
The TiME signature to predict response to immune checkpoint inhibitors. (**A**) Predictive role of the TiME signature in a mouse MPM model treated with anti–CTLA-4 antibodies. (**B**) AUC analysis of TiME signature in a mouse MPM model treated with anti–CTLA-4 antibodies. (**C**) Predictive role of the TiME signature in a cohort of patients with advanced melanoma treated with PD-1 blockade (*n* = 27). (**D**) Predictive role of the TiME signature in 10 unresectable human MPM patients treated with anti–PD-1 therapy. The 2-tailed Fisher’s exact tests were used to compare the data. CR, complete response; CTLA-4, cytotoxic T-lymphocyte associated protein 4; MPM, malignant pleural mesothelioma; mRECIST, modified response evaluation criteria in solid tumors; PD, progressive disease; PD-L1, programmed cell death 1 ligand 1; PD-1, programmed cell death 1; PR, partial response; SD, stable disease; and TiME, tumor immune microenvironment.

Considering the vastly different intratumoral immune environments of good-TiME and bad-TiME tumors and their differential expression of targets for checkpoint inhibitors (PD-1, PD-L1, and CTLA-4) between these immunologic subsets of MPM (Supplemental Figure 11), we hypothesized that such a signature could also predict response to checkpoint inhibitor therapy. We first examined publicly available mRNA expression data from a study in which immunocompetent mice were inoculated with bilateral s.c. murine AB1-HA mesothelioma tumors and treated with an anti–CTLA-4 antibody (42). All original microarray data were deposited in the NCBI’s Gene Expression Omnibus database (GEO GSE63557). Application of the TiME signature to this model identified 10 of 11 mice whose tumors responded to anti–CTLA-4 treatment (good-TiME) and 9 of 9 mice whose tumors did not respond (bad-TiME) (Figure 5, A and B). To evaluate TiME signature in human tumors, we analyzed mRNA sequencing data obtained from pretreatment tumor biopsies (GEO GSE78220) in metastatic melanoma patients treated with anti–PD-1 antibodies (*n* = 27) (43). Patients whose tumors demonstrated a good-TiME signature had statistically improved responses to PD-1 inhibition (Figure 5C). Finally, to evaluate utility of the TiME signature in MPM patients, we applied our TiME signature to the pretreatment biopsies (prior to immunotherapy) of 10 consecutive MPM patients with advanced and unresectable MPM whom we treated with anti–PD-1 therapy after they had progressed after treatment with a platinum-based agent and pemetrexed, in accordance with current National Comprehensive Cancer Network guidelines (44). Among 5 patients whose tumors demonstrated a good-TiME signature, 3 had a complete response by modified response evaluation criteria in solid tumors (mRECIST), 1 had partial response, and 1 had stable disease. The dramatic effects of the PD-1 inhibitor nivolumab in 1 patient with biphasic MPM (good-TiME) at 8 months after treatment is shown in Figure 5D, and chest CTs of the 2 additional complete responders are shown in Supplemental Figure 12. Among 5 patients whose tumors demonstrated a bad-TiME signature, 4 had progressive disease and 1 had stable disease. Further, the percentage or intensity of the PD-L1 clinical IHC test did not correlate with response to anti–PD-1 therapy (Figure 5D), and the TiME signature outperformed previously reported predictive immune signatures (45, 46) of response to PD-1 blockade (Supplemental Figure 13). Notably, mutational load, neoantigen burden, copy number alteration, and diversity of T cell clonality did not correlate with patients likely to respond to PD-1 blockade (i.e., good-TiME tumors) (Supplemental Figure 14). Thus, immunogenomic elements previously reported to correlate with response to checkpoint blockade were not present in MPM tumors that were likely to respond to PD-1 inhibition; however, these tumors could be identified by high abundance of neoantigens and, in particular, high abundance of neoantigens concomitant with high expression of their corresponding, specific MHC proteins.

## Discussion

Within the tumor microenvironment, a unique and multifaceted immune system encompasses elements such as innate and adaptive immune cells, stromal cells, cytokines and chemokines, and targets of checkpoint blockade that dictate clinical outcome and responses to immunotherapy (47, 48). As an emerging high-dimensional single-cell analysis platform, CyTOF is uniquely suited for investigating the complex intratumoral immune system by capturing the phenotypes and behavior of integrated cellular systems (13). We comprehensively characterized the intratumoral immune system of human MPM with CyTOF and applied unsupervised clustering of these data to identify a distinct immunogenic TiME signature that was associated with favorable OS and with response to checkpoint blockade. Tumors with a good-TiME signature were enriched for partially exhausted CD8^+^ T cells that have enhanced capacity to release IFN-γ, activated pDC, HLA-DR^+^ cancer cells, and decreased numbers of IL-10– and IL-17–releasing Tregs and PD-L1^+^ TAM. Proteomic analyses of these tumors with MS facilitated development of a simple gene signature that could be applied to all MPM tumors to determine their TiME profile.

Checkpoint inhibitors are changing the landscape of treatment for patients with solid tumors (49); however, less than half of all patients show favorable response (50–54), underscoring the unmet need for a clinical test that could predict response to these drugs. Whereas immunohistochemical expression of PD-L1 on tumors has been shown in some studies to correlate with response to checkpoint blockade (50–54), this is not true for all tumor types, and PD-L1 expression does not correlate with clinical response in a number of studies (50–54). In our experience in patients with advanced and unresectable MPM who were treated with anti–PD-1, the biomarker most predictive of response to anti–PD-1 therapy was the good-TiME signature. In contrast, PD-L1 immunohistochemical staining and previously reported immune signatures (45, 46, 55) did not correlate with therapeutic response. Application of this signature to a cohort of patients with advanced melanoma also demonstrated prediction of response to PD-1 inhibitors, suggesting a potential generalizability of this TiME signature to other solid tumors.

To eliminate cancer cells in the presence of immune checkpoint inhibitors, T cells must recognize antigens displayed by MHCs on tumor cells. Several clinical trials have suggested that the frequency of somatic mutations within a tumor type and, by extension, the potential for expression of tumor-specific neoantigens are correlated with sensitivity to immune checkpoint inhibitors. For example, in patients with melanoma, PD-1–expressing neoantigen-specific T cells have been identified in the peripheral blood and correlate with activity of PD-1 inhibitors (56). Similarly, in non–small cell lung cancer (NSCLC), anti–PD-1 therapy can induce neoantigen-specific T cell responses, and neoantigens have been associated with durable clinical benefits in patients with NSCLC and melanoma treated with checkpoint blockade (44). However, in many instances, tumor mutation burden and prediction of neoantigen burden do not correlate with response to checkpoint blockade (21, 25, 45, 46, 57). Whereas neoantigen burden has generally been defined based on epitope prediction algorithms using genomic and transcriptomic data (47), detection of neopeptides via MS is an innovative and evolving approach (27). We utilized MS to detect and quantify the abundance of potential neoantigens in MPM tumors and to quantify protein expression of their corresponding MHC-I and MHC-II HLA proteins. Our data demonstrated that high abundance of a neoantigen, in the presence of high expression of its corresponding MHC protein, underlies the strongest clinical responses to checkpoint blockade and highlights the importance of both neoantigen abundance and neoantigen presentation in clinical responses to these agents.

A limitation of our study is the small number of cases and exclusive number of surgical specimens used to generate the immunologic profile that was applied to both cohorts with earlier disease (surgical cohorts) and those with more advanced disease (treated with checkpoint blockade). However, the high fidelity of mass cytometry and simultaneous evaluation of more than 700 cellular subpopulations may compensate, to some extent, for our modest cohort sizes. Delineation of 2 distinct TiME profiles by comprehensive single-cell analyses demonstrated clinical utility in stratifying OS and in selection of patients for receipt of immunotherapy. Such a newly identified signature could potentially be applied to pretreatment tumor biopsies as a gene expression test or via a single-cell cytometry platform (41). Although prospective validation will be required before clinical adaptation, this signature may be useful for identifying patients who should undergo immune checkpoint blockade (good-TiME) and patients who should undergo alternative systemic therapy (bad-TiME).

In summary, a comprehensive, data-driven investigation of the intratumoral cellular immune system in MPM identified a distinct immunogenic tumor microenvironment that could be represented with a simple molecular signature prognostic for survival and prediction of response to checkpoint blockade. This work further proposes a potentially novel mechanism of response to checkpoint blockade in MPM that may be applicable to other human tumors: the requirement of a high abundance of neoantigens in the presence of high expression of MHC proteins specific for these neoantigens. Although prospective validation will be required before clinical adaptation, this signature may be useful for identifying patients who should undergo immune checkpoint blockade (good-TiME) and patients who should undergo alternative systemic therapy (bad-TiME).

## Methods

### Patient cohort.

A prospectively maintained single institution database was retrospectively reviewed. We enrolled 12 consecutive MPM patients who underwent macroscopic complete resection without preoperative treatment from August 2015 to December 2015 and had enough size of tumor to perform mass cytometry requiring at least 1 cm^3^ of tumor, MS requiring snap-frozen tissue, and mRNA microarray requiring snap-frozen tissue. We also tested our TiME signature to the preimmunotherapy tumor biopsies of 10 consecutive MPM patients with advanced and unresectable MPM, whom we treated with anti–PD-1 therapy after they had progressed after treatment with a platinum-based agent and pemetrexed between 2014 and 2016.

### Spanning-tree progression analysis of density-normalized events (SPADE) algorithm.

TiME was characterized using CyTOF with a 35-antibody panel (Supplemental Table 2) and SCAFFOLD maps (16) were generated. SPADE is a visualization tool that organizes heterogeneous populations of single-cell data into a 2-dimensional (2-D) tree representation based on similarities across user-selected markers (56, 57). The nodes of the tree represent clusters of cells that are similar in protein marker expression. SPADE uses the size and color of each node to denote the number of cells and median marker expression, respectively, thereby enabling users to quickly review a high-dimensional parameter space with a 2-D tree display. The branching structure of the tree, or the edge, can be used to infer cellular hierarchies when the tree is built using lineage-related surface markers. We used SPADE to perform density-dependent downsampling for each individual sample separately. We next applied the clustering step to the subset of the downsampled data comprising the overlapping core surface markers measured across downsampled cells in the 12 MPM samples and normal lung and pleura. The number of clusters was set to 500 because the increased number of markers could capture more cell types and branch points. Eventually, 742 nodes were generated from SPADE.

### SPADE-based SCAFFOLD map generation as mixture of human-guided knowledge and automated clustering.

Total live nucleated cells were used for all analyses. We defined 15 cellular phenotypes (Supplemental Figure 2): CD4^+^ T cells (CD45^+^CD3^+^CD4^+^), CD4^+^ Tregs (CD45^+^CD3^+^CD4^+^CD25^+^FOXP3^+^CD127^–^), CD8^+^ T cells (CD45^+^CD3^+^CD8^+^), partially exhausted CD8^+^ T cells (CD45^+^CD3^+^CD8^+^PD-1^+^CTLA-4^+^), monocytes (CD45^+^CD3^–^HLA-DR^+^CD14^+^CD11c^+^), TAM (CD45^+^CD3^–^HLA-DR^+^CD68^+^CD11b^+^CD11c^–^CD123^–^), pDCs (CD45^+^CD3^–^HLA-DR^+^CD11c^–^CD123^+^CD68^–^), cDC (CD45^+^CD3^–^HLA-DR^+^CD14^–^CD11c^+^), neutrophils (CD45^+^CD3^–^HLA-DR^-^CD15^+^CD56^–^), NK cells (CD45^+^CD3^–^HLA-DR^–^CD56^+^CD15^–^), cancer cells (CD45^–^Pan-cytokeratin^+^ [CD45^–^Pan-CK^+^]), cancer stem cells (CD45^–^Pan-CK^–^CD200^–^Vimentin^–^D24^+^CD326^+^ [CD45^–^Pan-CK^–^CD200^–^Vim^–^CD24^+^EpCAM^+^]), CAFs (CD45^–^Pan-CK^–^Vim^+^CD200^–^), mesothelial cells (CD45^–^Pan-CK^–^CD200^+^Vim^–^), and stromal cells (CD45^–^Pan-CK^–^CD200^–^Vim^–^CD24^–^CD326^–^). Among the 742 nodes generated from SPADE, a representative node in each phenotype was marked. To facilitate exploration by domain experts of deeper hierarchical inferences by annotating nodes on the tree as known cell types and thereby attributing directionality to specific branches or parts of a branch, cell populations and proteins were rearranged according to unsupervised average linkage hierarchical clustering of all nodes (Supplemental Figure 4). On the basis of the SPADE tree, newly generated hierarchical inferences were combined with the representative node. A cluster of similar pattern of protein expression was regarded as the same phenotype containing the representative node. After the arrangement of all nodes to phenotypes, their connection was displayed with SCAFFOLD maps. SCAFFOLD maps were generated as previously reported (16). Briefly, a graph was constructed by connecting the nodes representing the manually gated landmark populations and then connecting to them the nodes representing the cell clusters, as well as connecting the clusters to one another. Each node was associated with a vector containing the median marker values of the cells in the cluster (unsupervised nodes) or gated populations (landmark nodes). Edge weights were defined as the cosine similarity between these vectors after comparing the results from the implementation of several distance metrics. Each circle represented a node, a population of cells with the same pattern of expression, and the size of the node represented the percentage of cells in each patient. The clusters for all the tissues were combined in a single graph, with edge weights defined as the cosine similarity between the vectors of median marker values of each cluster. All the pairwise distances were calculated. The graph was then laid out using the ForceAtlas2 algorithm in Gephi 0.9.1 (https://gephi.org). In this representation of the cellular immune system of human MPM, each node was a population of cells with a similar pattern of protein expression, and the size of the node correlates with the average number of cells in the corresponding cell population. To overlay the additional samples on the SCAFFOLD map, the position and identity of the landmark nodes was fixed and the clusters of each sample were connected to the landmark nodes as described above.

To investigate the immunogenomic determinants of TiME, we investigated tumor mutational load, neoantigen burden, copy number alteration, diversity of T cell clonality, and neoantigen abundance. The experimental and analytical methods for integrated analyses of CyTOF, MS, and mRNA transcriptome data are available in the Supplemental Methods.

### Direct identification and quantification of mutated peptides using MS.

Next-generation sequencing data from the BWH cohort (33), the MSKCC cohort (34), and the COSMIC database (http://cancer.sanger.ac.uk/cosmic) (35) were used to generate a reference database containing 2,299 missense mutation sites in 1,885 proteins from 640 MPM tumors where a detected mutation alters the amino acid sequence (Supplemental Figure 7 and Supplemental Table 3). A peptide database containing a 29–amino acid length sequence, which includes point mutation site at the middle of the sequence, was generated. Obtained MS/MS spectra are searched against a customized human RefSeq database containing a mutated protein sequence obtained by DNA sequencing in the Proteome Discoverer 2.1 interface (Thermo Fisher Scientific) with Mascot algorithm (Mascot 2.4, Matrix Science). The peptides identified from the Mascot result file are validated with 5% FDR. An in-house intensity-based absolute quantification (iBAQ) algorithm and integrated peak alignment corrector (iPAC) are used to calculate protein abundance with optimal AUC estimates for the detected peptide peaks. In this way, neoantigen abundance — the intensity (amount) of each neoantigen — is quantified.

The MS raw files from proteome profiling was recalculated against mutated peptide database using Proteome Discoverer 1.4 (PD1.4) with Mascot search engine (2.4.1) with <5% FDR. The AUC from the peptide-containing mutated point was extracted from PD1.4 and used for relative quantification (Supplemental Table 4).

### HLA molecular typing.

Study samples were HLA genotyped by Luminex-based sequence-specific oligonucleotide typing assays (Supplemental Table 5). Genomic DNA was extracted from cells using the Qiagen EZ-1 machine. Intermediate resolution of all HLA loci (HLA-A, HLA-B, HLA-C, HLA-DRB1, HLA-DRB3, HLA-DRB4, HLA-DRB5, HLA-DQA1, HLA-DQB1, HLA-DPA1, and HLA-DPB1) were performed using the LABType SSO HD kits (One Lambda). Briefly, the DNA was amplified using primers that were specific for each of the 11 HLA loci. The PCR product was biotinylated and denatured to make single-stranded DNA. This DNA was then hybridized to a series of probes specific for nucleotide sequences that were used to define the HLA genes. Each probe was bound to a uniquely fluorescent coded Luminex bead. The mixture was then washed to remove any excess, unbound PCR product. The bound PCR product was labeled with streptavidin conjugated with a PE fluorescent tag (SAPE). The DNA-bead complexes were run on a Luminex 100 platform and analyzed using HLA-FUSION v3.5.6 software.

### Prediction of binding affinity of mutated neopeptides to MHC molecules.

We then applied standard prediction algorithms of the IEDB (http://www.iedb.org/) (36) for identification of HLA class I (37, 38) and class II predicted (39, 40) neopeptides on all missense mutations and measured the AUC in MS, representing the amount of measured peptide ligands, termed neoantigen abundance. According to HLA molecular typing from blood DNA in each patient, the binding affinities of peptides to MHC-I (HLA-A, HLA-B, and HLA-C) and MHC-II (HLA-DRB1/B3/B4/B5, HLA-DP, and HLA-DQ) were estimated.

To choose a MHC-I binding prediction method, the prediction method list box allowed us to choose from a number of MHC-I binding prediction methods: artificial neural network (ANN), stabilized matrix method (SMM), SMM with a peptide/MHC binding energy covariance matrix (SMMPMBEC), scoring matrices derived from combinatorial peptide libraries (Comblib_Sidney2008), Consensus, NetMHCpan, NetMHCcons, PickPocket, and NetMHCstabpan (37, 38). The list of peptides was filtered to include 8- to 14-mer peptides that bind to HLA-A, HLA-B, and HLA-C allotypes. IEDB Recommended was the default prediction method selection. Based on availability of predictors and previously observed predictive performance, this selection tried to use the best possible method for a given MHC molecule. Currently, for peptide/MHC-I binding prediction for a given MHC molecule, IEDB Recommended used the Consensus method consisting of ANN, SMM, and Comblib if any corresponding predictor was available for the molecule. Otherwise, NetMHCpan was used. This choice was motivated by the expected predictive performance of the methods in decreasing order: Consensus > ANN > SMM > NetMHCpan > Comblib.

To choose a MHC-II binding prediction method, the prediction method list box allowed us to choose between 7 currently implemented MHC class II binding prediction methods: IEDB Recommended, Consensus method, Comblib, NN-align (netMHCII-2.2), SMM-align (netMHCII-1.1), Sturniolo, and NetMHCIIpan (39, 40). The list of peptides was filtered to include 15-mer peptides that bind to HLA-DRB1, HLA-DRB3, HLA-DRB4, HLA-DRB5, HLA-DPA1, HLA-DPB1, HLA-HQA1, and HLA-DQB1 allotypes. The default selection IEDB Recommended was provided. Based on availability of predictors and previously observed predictive performance, this selection tried to use the best possible method for a given MHC molecule.

To consider HLA binding strength relative to its nonmutant (WT) counterpart, peptides with IEDB percentile rank ≤2% affinity to mutant peptide sequence and rank >2% affinity to WT peptide sequence were regarded as potential binders to MHC-I. Among them, peptides with IEDB percentile rank ≤1% affinity to mutant peptide sequence and rank >1% affinity to WT peptide sequence were regarded as those with high affinity to MHC-I, and the others were regarded as those with intermediate affinity to MHC-I (Supplemental Table 6). Peptides with IEDB percentile rank ≤10% affinity to mutant peptide sequence and rank >10% affinity to WT peptide sequence were regarded as potential binders to MHC-II. Among them, peptides with IEDB percentile rank ≤5% affinity to mutant peptide sequence and rank >5% affinity to WT peptide sequence were regarded as those with high affinity to MHC-II, and the others were regarded as those with intermediate affinity to MHC-II (Supplemental Table 7).

### Statistics.

Student’s *t* tests, paired *t* tests, χ^2^ tests, and Fisher exact tests were used to compare the data. Survival curves were generated with the Kaplan-Meier’s method, and intergroup comparisons were performed with the log-rank test. OS was defined as the time from surgery to death. Data were censored when a patient was alive without recurrence at last follow-up. Univariable Cox regression analysis was used to determine what factors were associated with OS. We used multivariable Cox proportional hazards regression analysis to evaluate variables with *P* values less than 0.2 in univariable analysis (Supplemental Table 9). Statistical significance was accepted for *P* < 0.05, and all tests were 2-tailed. Biometric Research Branch (BRB) Array Tools were used for statistical analysis of the gene-expression data, and receiver operating characteristics (ROC) and all other statistical analyses were performed with SPSS 24.0 (SPSS Inc.) and R language and software environment (http://www.r-project.org).

### Study approval.

This study was performed in accordance with an IRB protocol at Baylor College of Medicine (H-36302). All human samples were collected with informed patient consent.

## Author contributions

HSL and BMB conceptualized and planned the study. HSL, HJJ, BMB, and DJS contributed to collection of surgical samples and associated clinical information. JZ administered immunotherapy to MPM patients. VVLDR conducted radiology assessment. TMW conducted pathology assessment. HSL, HJJ, JMC, SYJ, and BMB coordinated the data generation and data analysis. HSL and HJJ helped to generate mRNA data from TCGA database. HSL helped to generate mRNA sequencing data from BWH database. HSL and HJJ generated the gene expression data. JMC and SYJ generated the MS data. TT, PTJ, and RHK generated HLA molecular typing. HSL, HJJ, and JSL processed, analyzed, and participated in discussions related to the genomics data with statistics. HSL, HJJ, JZ, JMC, VR, TW, PTJ, JSL, SYJ, FK, DJS, and BMB participated in discussions and provided critical scientific input, analysis suggestions, and logistical support toward the project. HSL and BMB wrote the manuscript.

## Supplementary Material

Supplemental data

Supplemental Table 1

## Version 1 04/05/2018

Electronic publication
