# Supplementary material for: Comprehensive immunoproteogenomic analyses of malignant pleural mesothelioma
Source: JCI Insight. 2018 Apr 5;3(7):e98575. doi: 10.1172/jci.insight.98575 (PMC5928857; doi:10.1172/jci.insight.98575)
Supplement: Supplemental data [file jciinsight-3-98575-s001.pdf]

Supplementary Materials for

Comprehensive immunoproteogenomic analyses of malignant pleural mesothelioma

Hyun-Sung Lee, Hee-Jin Jang, Jong Min Choi, Jun Zhang, Veronica V Lenge De Rosen, Thomas M. Wheeler, Ju-Seog Lee, Thuydung Tu, Peter T. Jindra, Ronald H. Kerman, Sung Yun Jung, Farrah Kheradmand, David J. Sugarbaker, Bryan M. Burt

correspondence to: Bryan.Burt@bcm.edu

This PDF file includes:

Materials and Methods
Supplementary Figures 1 to 14
Supplementary Tables S1 to S9
Reference (58-97)

Other Supplementary Materials for this manuscript includes the following:

Tables S3, S6, and S7 as separate Excel files

Materials and Methods

Patients follow-up

Patients were evaluated preoperatively with chest CT, PET-CT, chest MRI, echocardiogram, pulmonary function tests, and V/Q scans. A staging procedure including cervical mediastinoscopy and diagnostic laparoscopy with peritoneal washings was performed to rule out mediastinal nodal disease and intraabdominal disease, retrospectively. Extrapleural pneumonectomy (EPP) was performed through an extended posterolateral thoracotomy and included resection of the lung, pericardium, and diaphragm (*58*). Extended pleurectomy/decortication (P/D) incorporated total visceral and parietal pleurectomy with resection of the pericardium and diaphragm (*59*). In both cases, the diaphragm and pericardium were reconstructed with soft tissue polytetrafluorethylene patch (Gore-Tex® Inc., Flagstaff, AZ). EPP was performed in cases in which complete macroscopic resection could not be achieved by P/D. Heated intraoperative chemotherapy (HIOC) with a 1 hour 41℃ bath of cisplatin was administered to patients with acceptable renal function (EGFR>60ml/min/1.73m^2). Following resection, patients underwent radiologic surveillance by chest CT with IV contrast every 4 months. Lesions suspicious for recurrence were further investigated with PET-CT and/or needle biopsy.

Determination of the number of samples

Power calculations were performed to derive a sample size of 12 which would allow detection of significant differences in protein expression of each cell population (node) that was measured by CyTOF. The sample size and power calculations were determined in a way considering the difference in fold change under log2 protein expression with a standard deviation of 0.7 according to our own experimental data (*60*). To detect two-fold protein expression change with 80% power at a multiple testing corrected type I error rate of 0.05 with 600 anticipated number of nodes with undifferentially expressed protein among total 742 nodes, a total of 12 samples were needed. Further, given the rarity of MPM, and the sample sizes of CyTOF analyses of human tumors in recently published reports (*12, 61, 62*), a sample size of 12 seems reasonable and comparable. For example, in lung cancer, which has much higher incidence than MPM, CyTOF data from 18 patients with lung adenocarcinoma were reported (*12*). Similarly, 12 hepatocellular carcinoma samples were evaluated (*61*). Additionally, in AML, 16 samples from cryopreserved diagnostic bone marrow mononuclear cells of pediatric AML patients were analyzed (*62*).

Time-of-flight mass cytometry (CyTOF)

Sample Collection

All human samples were delivered from the operating room to the research laboratory immediately after the specimens were resected. The samples were kept in RPMI without glutamate media in ice during transport. Cancer specimens were processed into single cell suspensions, fresh frozen tissue preparations, samples cryopreserved in optimal cutting temperature (OCT) compound, and formaldehyde fixed paraffin embedded tissues (FFPE). For CyTOF, single-cell suspensions were selected from 12 patients who were diagnosed with treatment-naïve MPM. Single cell suspensions of normal lung and normal pleura were obtained from a healthy individual undergoing surgery for a pneumothorax. Tissues from patients undergoing CyTOF also underwent mass spectrometry (MS) and mRNA microarray.

To predict response to anti-PD-1 therapy, FFPE tissues were obtained from an additional 10 patients with advanced, unresectable MPM who were treated with anti-PD-1 therapy.

Single cell preparations from human MPM tumors

The tumor specimens were finely minced and digested in unsupplemented RPMI 1640 (without glutamate) with a human Tumor Dissociation Kit (Miltenyi Biotec Inc., Auburn, CA, USA, Cat.No.130-095-929) in 50 ml Falcon tube for 30 minutes in the 37℃ rotating incubator. The cells were then filtered through a 70 µm cell strainer (Corning Life Sciences Plastic, Cat.No.07201431), washed, and lysed in ACK lysing buffer (Life Technologies, Cat.No.A049201). After centrifuging cells with unsupplemented RPMI media for 5 min at 400×*g* in 20℃ and washing two times, the supernatants were carefully suctioned off. The cell pellet underwent a final washing with 40 ml supplemented cell culture media (RPMI with FBS), and cells were refiltered through a 70 µm cell strainer. After centrifuging the cells for 5 min at 400×*g* in 20℃, the supernatant was carefully suctioned off. Some cells were placed in freezing media (FBS with 7% DMSO) and cryopreserved in -80℃ freezer storage after cell counting with cell counter (Countess II FL, Life Technologies, Cat.No.AMQAF1000). For long-term preservation, the cryovials were transferred into liquid nitrogen tank in -196℃.

CyTOF

Antibodies were chosen to facilitate the identification of immune cell types, stromal cells, and cancer cells. All mass cytometry antibodies (surface/cytokine/phosphoprotein) used in this study are listed in **Supplementary Table 2**. Antibodies were either purchased pre-conjugated from Fluidigm (http://maxpar.fluidigm.com/product-catalog-metal.php) or purchased purified and conjugated in-house using MaxPar® X8 Polymer Kits (Fluidigm) according to the manufacturer's instructions by the University of Texas MD Anderson Cancer Center Cytometry Core Facility. Surface and intracellular antibody cocktails were prepared for the staining of all samples for CyTOF.

Cryopreserved single cell preparations are stabilized for 6 hours in 37℃ incubator after thawing. Five minutes before antibody staining, Cell ID™-cisplatin (MaxPar®) was added to assess cell viability. Dead cells were stained with Cell-ID Cisplatin according to the manufacturer's protocol. Five hundred thousand cells were resuspended in Maxpar® Cell Staining Buffer (Fluidigm, Cat.No.201068) in individual 5 mL tubes for each sample to be barcoded. Mass-tag cellular barcoding using the Cell-ID 20-Plex Pd Barcoding Kit (Fluidigm®, Cat.No. 201060) was performed as previously described (*63*). Briefly, 0.5 x 10^6 cells from each sample were barcoded with distinct combinations of stable palladium (Pd) isotopes chelated by isothiocyanobenzyl-EDTA in 0.02% saponin in PBS. After washing, cells were resuspended in 1 mL Fix I Buffer (Fluidigm, Cat.No.201065), and incubated for 10 minutes at room temperature (RT). After washing twice with 1 mL of Barcode Perm Buffer (Fluidigm, Cat.No.201057), each sample was resuspended to be barcoded completely in 800 µL Barcode Perm Buffer. Barcodes were resuspended completely in 100 µL Barcode Perm Buffer and transferred to the appropriate samples. After mixing the sample immediately and completely, the samples were incubated for 30 minutes at RT. After washing twice with 1 mL of Maxpar® Cell Staining Buffer, the samples were resuspended in 100 µL Maxpar® Cell Staining Buffer, and all barcoded samples were combined into one tube.

For the first cohort of 12 MPM patients, cells were washed and incubated with extracellular antibodies for 30 minutes at RT and washed before being fixed and permeabilized in 1× Fix I buffer. The samples were then stained with intracellular antibodies for 30 minutes at RT and washed. Ten minutes before finishing staining with intracellular antibodies, 0.125 nM Cell-ID™ Intercalator-Ir (Fluidigm®, Cat.No.201192B) in Maxpar® Fix and Perm Buffer (Fluidigm®, Cat.No.201067) was added. Cell-ID™ Intercalator-Ir is a

cationic nucleic acid intercalator that contains naturally abundant Iridium (191Ir and 193Ir) and is used for identifying nucleated cells in CyTOF analysis.

For the second experiment analyzing 4 epithelioid MPM patient samples to evaluate the functional role of major cellular phenotypes, cells from each patient were split into 6 fractions for a total of 24 aliquots. The 12 aliquots from each patient were incubated in 1 µl/mL Golgistop (BD cell analysis, Cat.No.BDB554724A), 2 µl/mL PMA/Ionomycin (eBioscience Cell Stimulation Cocktail, Cat.No.00-4970-93), and 5 µg/mL polyI:C (Poly(I:C) (HMW) VacciGrade™, InVivoGen, Cat.No.31852-29-6) for 6 hours at 37℃, according to standard protocol. The samples were then washed and incubated with cell surface antibodies for 30 minutes at RT and washed. After overnight at 4℃ with resuspension in 1× Fix I buffer, the samples were stained with intracellular antibodies against cells cytokines and phosphoproteins for 30 minutes at RT and washed. Ten minutes before finishing staining with intracellular antibodies, 0.125 nM Cell-ID™ Intercalator-Ir in Maxpar® Fix and Perm Buffer was added. The other 12 aliquots from each patient were fixed within 20 minutes after adding 2 µl/mL PMA/Ionomycin (eBioscience Cell Stimulation Cocktail, Cat.No.00-4970-93), and 5 µg/mL PolyI:C (Poly(I:C) (HMW) VacciGrade™, InVivoGen, Cat.No.31852-29-6) to evaluate phosphoproteins. Samples were washed and incubated with extracellular antibodies for 30 minutes at RT and washed. After adding 1 mL of 4℃ methanol to each sample for 15 minutes on ice, the samples were stained with phosphoprotein antibody cocktail for 30 minutes at RT and washed. Ten minutes before finishing staining with intracellular antibodies, 0.125 nM Cell-ID™ Intercalator-Ir in Maxpar® Fix and Perm Buffer was added.

CyTOF Data Acquisition

After washing cells with PBS and MilliQ water, stained cells were analyzed on a mass cytometer (CyTOF2™ mass cytometer, Fluidigm) at an event rate of 400 to 500 cells per second. Data files for each sample were normalized with Normalizer v0.1 MCR and gated. The bead standards were prepared immediately before analysis, and the mixture of beads and cells were filtered through a filter cap FACS tubes before analysis. All mass cytometry files were normalized together using the mass cytometry data normalization algorithm (*64*), which used the intensity values of a sliding window of these bead standards to correct for instrument fluctuations over time and between samples. Barcodes were deconvoluted using the Debarcoder® software (Fluidigm®) (*63*).

CyTOF Data Analysis

The schema of CyTOF data analysis is illustrated in **Supplementary Figure 1**. Fifteen cellular phenotypes were manually defined by a panel of 35 antibodies (**Supplementary Figure 2**) and pooled data from the 12 MPM patients incorporated into a SCAFFOLD map containing 742 nodes that were generated by the SPADE analysis, a density-based algorithm for visualizing single-cell data and enabling cellular hierarchy inference among subpopulations of similar cells.

Spanning-tree Progression Analysis of Density-normalized Events (SPADE) algorithm

SPADE is a visualization tool that organizes heterogeneous populations of single-cell data into a 2-dimensional (2D) tree representation based on similarities across user-selected markers (*65, 66*). The nodes of the tree represent clusters of cells that are similar in protein marker expression. SPADE uses the size and color of each node to denote the number of cells and median marker expression, respectively, thereby enabling users to quickly review a high-

dimensional parameter space with a 2D tree display. SPADE makes use of density-dependent down-sampling followed by agglomerative clustering in order to prevent rare cells from being overlooked among the more abundant cellular populations. Briefly stated, SPADE is composed of four major steps: (i) density-dependent down-sampling, (ii) agglomerative clustering, (iii) linking clusters with a minimum spanning-tree algorithm, and (iv) up-sampling to map all cells onto the final output tree. SPADE is available at http://pengqiu.gatech.edu/software/SPADE/. The branching structure of the tree, or "the edge", can be used to infer cellular hierarchies when the tree is built using lineage-related surface markers. Because SPADE uses the minimum spanning-tree algorithm, its output is unrooted and does not prescribe the direction for hierarchical assessment.

We used SPADE to perform density-dependent down-sampling for each individual sample separately. We next applied the clustering step to the subset of the down-sampled data comprising the overlapping core surface markers measured across down-sampled cells in the 12 MPM samples and normal lung and pleura. The number of clusters was set to 500 because the increased number of markers could capture more cell types and branch points. Eventually 742 nodes were generated from SPADE.

SPADE-based SCAFFOLD (Single-Cell Analysis by Fixed Force- and Landmark-Directed) map generation as mixture of human guided knowledge and automated clustering

Total live nucleated cells were used for all analyses. We defined 15 cellular phenotypes (**Supplementary Figure 2**): CD4 T cells (CD45^{+}CD3^{+}CD4^{+}) (*65, 67*), regulatory CD4 T cells (Tregs; CD45^{+}CD3^{+}CD4^{+}CD25^{+}FOXP3^{+} CD127^{-}) (*65, 67, 68*), CD8 T cells (CD45^{+}CD3^{+}CD8^{+}) (*65, 67, 69*), partially exhausted CD8 T cells (CD45^{+}CD3^{+}CD8^{+}PD-1^{+}CTLA-4^{+}) (*69-72*), monocytes (CD45^{+}CD3^{-}HLA-DR^{+}CD14^{+}CD11c^{+}) (*65, 67*), tumor-associated macrophages (TAM; CD45^{+}CD3^{-}HLA-DR^{+}CD68^{+}CD11b^{+} CD11c^{-}CD123^{-}) (*65, 67, 73*), plasmacytoid dendritic cells (CD45^{+}CD3^{-}HLA-DR^{+}CD11c^{-}CD123^{+}CD68^{-}) (*65, 67, 74*), conventional DC (CD45^{+}CD3^{-}HLA-DR^{+}CD14^{-}CD11c^{+}) (*65, 67, 74*), neutrophils (CD45^{+}CD3^{-}HLA-DR^{-}CD15^{+}CD56^{-}) (*65, 67*), NK cells (CD45^{+}CD3^{-}HLA-DR^{-}CD56^{+}CD15^{-}) (*65, 67*), cancer cells (CD45^{-}Pan-cytokeratin (CK)$^{+}$) (*75, 76*), cancer stem cells (CD45^{-}Pan-CK^{-}CD200^{-}Vimentin(Vim)$^{-}$CD24^{+}CD326(EpCAM)$^{+}$) (*77-81*), cancer-associated fibroblasts (CAFs; CD45^{-}Pan-CK^{-}Vim^{+}CD200^{-}) (*75, 82, 83*), mesothelial cells (CD45^{-}Pan-CK^{-} CD200^{+}Vim^{-}) (*84-86*), and stromal cells (CD45^{-}Pan-CK^{-}CD200^{-}Vim^{-}CD24^{-}CD326^{-}). We used a general marker of cancer stem cells CD326 (*77, 78*) and mesothelioma specific cancer stem cell marker CD24 (*79-81*). Cancer stem cells were gated as CD45- Pan-CK- CD200-Vim-CD24+ CD326+. In addition, we confirmed that normal pleural tissue is mainly composed of mesothelial cells with CD45-PanCK-CD200+ Vim- based on several references (84-*86*) (**Supplementary Figure 3**).

Statistical Comparison of Two TiME subsets

Cells from each tissue sample, for all patients, were clustered together. Cells were then deconvoluted into their respective samples via their barcoded identifier. Cell frequencies were calculated as a percent of total live nucleated cells (excluding erythrocytes). Cell frequency of less than 0.01% was filtered out. Cluster frequencies for each cluster were passed into the Significance Across Microarrays algorithm (*14, 61, 87*) through BRB-Array Tools version 4.3.0 (Biometric Research Branch, National Cancer Institute, Bethesda, MD, USA) and results were tabulated into the SCAFFOLD map files for visualization through the graphical user interface. We next performed unsupervised clustering (*16, 66*) of all nodes according to % cell frequency of total live nucleated cells, which revealed two distinct patterns, named TiME-I and TiME-II. We compared TiME-I and TiME-II to determine the

significant internodal differences between two subsets. Among 742 nodes, 50 nodes showed significant internodal differences between TiME-I and-II (False Discovery Rate <0.35). Next, to generate the statistical values of phenotypic differences between two subsets, the internodal differences in the same phenotypes were analyzed with paired t-test according to the corresponding nodes. SCAFFOLD maps were then generated as previously reported (*15*).

For the functional investigation of two subtypes to compare co-stimulatory or co-inhibitory molecules between two TiME subsets, we analyzed the expression of 35 markers in different proportions in two subsets (**Figure 2D**). Z ratios were calculated by taking the difference between the averages of the observed marker Z scores and dividing by the standard deviation of all of the differences for that particular comparison. A Z ratio of ±1.96 was inferred as significant ($P<0.05$) (*88*).

CyTOF data was additionally analyzed with FlowJo V10 software to confirm consistency of results by CyTOF analysis (**Supplementary Figure 5A** and **Supplementary Figure 6**). Median fluorescent intensities (MFI) of cytokines and phosphoproteins were compared with t-tests according among phenotypes between TiME-I and TiME-II. MFIs between partially exhausted CD8 T cells and the other CD8 T cells, Tregs cells and the other CD4 T cells, cDC and pDC, PD-L1 positive and negative TAMs, HLA-DR$^+$ and HLADR$^-$ cancer cells were compared, respectively. Such cellular alterations were analyzed in a similar fashion before and after stimulation of PMA/Ionomycin and PolyI:C (**Supplementary Figure 5B**).

Protein profiling and Direct Identification of neopeptides by Mass Spectrometry

Sample digestion and peptide extraction

MPM tissues were kept in -80℃ until further processing. Frozen tissues were cryogenically ground, dispersed by pipetting in lysis buffer (50 mM Ammonium Bicarbonate, 1 mM $CaCl_2$) and then snap frozen using liquid nitrogen and thawed at 37℃. Proteins were then boiled at 95℃ for 3 min. All freeze-thaw-denaturation procedures were repeated three times. Protein concentration was measured using Brad-ford reagent and 40 µg proteins were digested with 2 µg of trypsin (GenDepot, T9600) for O/N at 37°C. After the first digestion, an additional 400 ng of trypsin was added to the samples, which was then incubated for 4 h at 37℃. Double-digested peptides were extracted by 50% Acetonitrile/0.1% formic acid and peptide supernatant was taken after spin-down at 10,000 ×*g* for 1 min. The remaining pellet was extracted with 80% acetonitrile/0.1% formic acid once again and pooled into previous extract after spinning down. Pooled peptide supernatant was measured for peptide concentration by colorimetric assay (Pierce, 23275). Peptide aliquots (40 µg) were made and dried using vacuum drier and stored at -20℃ until further procedures.

High pH C18 reverse phase sample preparation

Vacuum dried peptides were dissolved in pH10 buffer (10 mM Ammonium Bicarbonate, adjusted to pH of 10 by NH_4OH) and subjected to pH10 C18 reverse phase column chromatography. A micro pipet tip C18 column was made from 200 µl pipet tip by layering 6 mg of C18 matrix (Reprosil-Pur Basic C18, 3µm) on top of the C18 disk (3M, Empore™ C18) plug. Vacuum-dried peptides were dissolved with 150 µl of pH10 buffer and loaded on the C18 tip equilibrated with pH10 solution. Bound peptide was eluted with step gradient of 100 µl of 6, 9, 12, 15, 18, 21, 25, 30, 35% ACN (pH10) and pooled into 6 pools (6% eluent combined with 25% eluent, 9% plus 30%, and 12% plus 35%) and vacuum dried for nanoHPLC-MS/MS.

Nano HPLC-MS/MS analysis

Vacuum dried peptides were dissolved in 10 µl of loading solution (5% methanol containing 0.1% formic acid) and one half of the reconstituted sample was subjected to nanoLC-MS/MS analysis with an Ultimate 3000 UPLC (Thermo Scientific) coupled to Thermo Fusion (Thermo Scientific) mass spectrometer. The peptides were loaded onto an in-house Reprosil-Pur Basic C18 (1.9 µm, Dr.Maisch GmbH, Germany) trap column of 2 cm × 100 µm size. Then the trap column was washed with loading solution and switched in-line with an in-house 6 cm × 150 µm column packed with Reprosil-Pur Basic C18 equilibrated in 0.1% formic acid/water. The peptides are separated with a 75 min discontinuous gradient of 2-24, 4-24 or 8-26% acetonitrile/0.1% formic acid at a flow rate of 850 nl/min. Separated peptides were directly electro-sprayed into mass spectrometer. The instrument was operated in data-dependent mode, acquiring fragmentation spectra of the top 50 strongest ions and under direct control of Xcalibur software (Thermo Scientific). Parent MS spectrum and HCD fragmented MS/MS spectrum were acquired in the Orbitrap and Ion trap with resolution of 120,000 and rapid scan speed respectively. The full MS range was 375-1300 m/z and the trap target was 500,000. MS/MS ion target value was 5,000 ions.

Protein Peptide identification and label-free quantification

Obtained MS/MS spectra were searched against target-decoy human refseq database (release 2015_06, containing 58,549 entries) in the Proteome Discoverer 1.4 interface (PD1.4, Thermo Fisher) with the Mascot algorithm (Mascot 2.4, Matrix Science). Dynamic modifications of Acetylation of N-term and Oxidation of methionine were allowed. The precursor mass tolerance was confined within 20 ppm with fragment mass tolerance of 0.5 Da and a maximum of two missed cleavages was allowed. Assigned peptides were filtered with 1% false discovery rate (FDR) using percolator validation based on q-value. iBAQ algorithm was used to calculate protein abundance. The Spectral assignments from PD1.4 were then converted to the MS-platform independent mzXML format and channeled through an in-house pipeline for peptide quantification (iPAC) and protein identification and quantification (grouper, which utilizes iPAC results). iPAC (integrated Peak Alignment Corrector) is a program to used obtain optimal area under-the-curve (AUC) estimates for the detected peptide peaks, which extracted candidate peptide information from the searching result list, including peptide, protein ID, modification, charge, m/z, retention time (RT), and scan number. These intensity values could be constructed into Extracted Ion Chromatogram (XIC) peak for the peptide along the RT axis. Overall, this approach allows researchers to visualize not only quantitative, but also qualitative differences in protein measurements with ease (*89*).

<u>Correlation between abundance of peptides with high affinity to MHC molecules and the expression of MHC molecules according to TiME subsets</u>

To evaluate the distribution of neoantigen abundance of high-affinity peptides and MHC molecules between two TiME subsets, we performed chi-square or Fisher's exact tests in four quadrants divided by median value of each axis. Further comparison was then performed between the right upper quadrant (high neoantigen abundance and high expression of MHC proteins) with the other quadrants. This revealed that both higher expression of HLA-A, HLA-B, HLA-DRB1, and HLA-DP (**Supplementary Figure 8**) and higher neoantigen abundance of their binding peptides are significantly associated with TiME-I tumors. In 2D

plots, the vertical dot line is the median value of MHC molecules, and horizontal dot line is the median value of neoantigen abundance (**Supplementary Figure 9**).

Prediction Model with TiME Signature Determined from Proteome and Transcriptome Analysis

Total RNA from fresh frozen tissues was extracted with MirVana (Invitrogen, Carlsbad, CA, USA) according to the manufacturer's protocols. RNA quality was assessed with an Agilent 2100 bioanalyzer and the RNA 6000 NanoChip kit (Agilent Technologies, Santa Clara, CA), and RNA quantity was determined using an ND-2000 spectrophotometer (NanoDrop Technologies, Wilmington, DE, USA). FFPE samples were sliced to 10 μm thickness, and 3-5 slices were put into a 1.5-ml tube. After deparaffinization with xylene, total RNA was extracted with a RecoverAll Total Nucleic Acid Isolation Kit (Ambion, Austin, TX, USA), according to the manufacturer's instructions. RNA quality and quantity were assessed with the aforementioned methods.

To identify the mRNA expression profile of MPM samples, mRNA microarray experiments were performed with a HumanHT-12 v4 Expression Beadchip Kit (Illumina, San Diego, CA, USA). Using a TotalPrep RNA Amplification Kit (Illumina), we labeled and hybridized 750 ng of total RNA according to the manufacturer’s protocols. After beadchips were scanned with a BeadArray Reader (Illumina), microarray data were analyzed with the Robust MultiArray Average algorithm and implemented quantile normalization with log2 transformation of gene expression intensities with BRB-Array Tools version 4.3.0 and the R-script from the Bioconductor project (www.bioconductor.org). Then, we selected the human mRNAs and adjusted data with median values for genes and arrays, respectively. An unsupervised hierarchical clustering algorithm was applied using the uncentered correlation coefficient as the measure of similarity and the method of average linkage (Cluster 3.0). Java Treeview 1.60 (Stanford University School of Medicine, Stanford, CA, USA) was used for tree visualization. Microarray data have been deposited in the National Center for Biotechnology Information’s (NCBI) Gene Expression Omnibus (GEO): GSE99070.

Integrated analyses of proteins obtained from mass spectrometry (MS) and mRNAs from mRNA arrays were performed to identify a gene signature from MPM tumors that could discriminate TiME-I and TiME-II tumors. Among 2944 mRNA with differential expression ($P<0.05$) between TiME-I and TiME-II, we selected 137 mRNAs whose expression was also statistically different between TiME-I and TiME-II cohorts at the protein levels, and which defined our TiME signature (**Supplementary Table 8**). Analyses of this signature in other datasets was performed following data extraction of mRNA sequencing data of the 211 samples in BWH cohort (*33*) from European Genome-phenome Archive under accession code EGAS00001001563, 69 samples in TCGA cohort (https://gdc-portal.nci.nih.gov/), mRNA gene expression data from the 50 samples in MSKCC cohort (MSKCC; GSE29211) (*34*), mouse mRNA gene expression microarrays (GSE63557) (*42*), and human melanoma mRNA gene expression microarray (GSE78220) (*43*) in NCBI GEO (**Supplementary Table 1**). To predict the TiME subsets in each samples in the independent cohorts, we adopted a previously developed model (*60, 87*). Briefly, TiME signature expression data were used to generate a compound covariate predictor (CCP) (*91*) classifier for estimating the likelihood that a particular MPM tumor belonged to the subgroup in which the TiME signature is present (TiME-I subgroup) or the subgroup in which the signature is absent (TiME-II subgroup). Data in the training cohorts were combined to form a series of classifiers, and the robustness of the classifier was estimated by the misclassification rate determined during leave-one-out cross-validation (LOOCV) of the training set. After LOOCV, the sensitivity

and specificity of the prediction models were estimated by the fraction of samples correctly predicted (**Supplementary Figure 10**).

Expression of immune co-inhibitory molecules in MPM

Representative immune co-inhibitory molecules such as PD-1, CTLA-4, and PD-L1 were evaluated in MPM subsets and compared with normal lung and pleura (**Supplementary Figure 11**). We found that PD-1, CTLA-4, and PD-L1 were elevated in MPM tumors and that normal pleura did not express them. Normal lung demonstrated very low expression of PD-1 and CTLA-4 in CD45+ immune cells, but high expression of PD-L1 likely due to its expression on alveolar macrophages. Tumors in TiME-I MPM had high expression of PD-1, CTLA-4 and PD-L1 on immune cells, and low expression of PD-L1 on non-immune cells. In contrast, TiME-II tumors demonstrated lower expression of PD-1 and CTLA-4 on immune cells, and high expression of PD-L1 on tumor cells and immune cells.

Performance of the TiME signature to predict response to anti-PD-1 therapy for MPM

The IFN-ɣ signature (CIITA, GBP4, GBP5, IRF1, IRF2, and JAK2) and immune signature (CD2, CD247, CD3E, GZMH, GZMK, NKG7 and PRF1) (*45*), cytolytic activity (GZMA and PRF1) (*46*, immunophenoscore in The Cancer Immunome Atlas (TCIA) (55) have been reported to predict responses to immune checkpoint inhibitors in human malignancies including melanoma. We tested the performance of the TiME signature to predict the response to anti-PD-1 therapy in patients with MPM alongside these other immune signatures. The average of the expression value of all the genes in the corresponding signature was utilized for these analyses (**Supplementary Figure 13**).

Immunogenomic analyses

To investigate the immunogenomic determinants of TiME, we studied mutational load (*92*), neoantigen burden (*21-22*), copy number alteration (*38*, *93-95*), and diversity of T cell clonality (*96*, *97*) (**Supplementary Figure 14**).

Mutational load from the TCGA

Mutational data (BCGSC_IlluminaHiSeq_DNASeq_automated) downloaded from the TCGA (https://portal.gdc.cancer.gov/) were analyzed (n=82). The number of nonsense mutation and missense mutation was compared between good-TiME and bad-TiME.

Predicted neoantigen burden from the BWH cohort

Predicted neoantigen burden to HLA-A in BWH cohort (n=95) was downloaded (*33*) and compared between good-TiME and bad-TiME tumors.

Copy number alteration analysis from the TCGA.

Copy number segments of log2 copy ratios (tumor/normal) for each tumor sample was obtained from http://firebrowse.org/#. GISTIC (*98*) identifies genomic regions that are significantly gained or lost across a set of tumors. The pipeline first filtered out normal samples from the segmented copy-number data by inspecting the TCGA barcodes and then executed GISTIC version 2.0.22. There were 87 tumor samples used in this analysis: 21 significant arm-level results, 0 significant focal amplifications, and 21 significant focal deletions were found. Copy number amplifications were regions with a log2 ratio above 0.1

and were considered amplified. As a threshold for copy number deletions, regions with a log2 ratio below the negative of 0.1 were considered deletions. In all thresholds by genes (all_thresholded.by_genes.txt in CopyNumber Gistic2 in CopyNumber Analyses of mesothelioma (MESO) from http://firebrowse.org/#), a gene-level table of discrete amplification and deletion indicators was developed for all samples. There was a row for each gene. A value of 0 meant no amplification or deletion above the threshold. Amplifications were positive numbers: 1 meant amplification above the amplification threshold; 2 meant amplifications larger to the arm level amplifications observed for the sample. Deletions were represented by negative table values: -1 represented deletion beyond the threshold; -2 meant deletions greater than the minimum arm-level deletion observed for the sample. It was used to identify copy number gain (log2 copy ratios > 0.1) or loss (log2 copy ratios < −0.1) at the gene level. The burden of copy number gain or loss was then calculated as the total number of genes with copy number gain or loss per sample.

Diversity of T cell clonality from the TCGA

Li et al. developed a novel computational method for de novo assembly of CDR3 regions using paired-end RNA-seq data, and applied it to 9,142 samples from the Cancer Genome Atlas (TCGA) (*96*). Clonotype diversity of T cell repertoire is an important property of the immune system and is closely related to the capacity for T cells to recognize antigens. As each T cell clone possesses a unique TCR, CDR3 sequences are often used as proxies to represent clonotype diversity. In their data, the number of unique CDR3 calls in each tumor is linearly correlated with total TCR reads, an expected observation since tumors with higher T-cell infiltrates have more TCR reads to assemble more CDR3 sequences. The number of unique CDR3 calls in each sample normalized by the total read count in the TCR region is called clonotypes per kilo-reads (CPK), as a measure of clonotype diversity. CPK values were compared between good- and bad-TiME within the TCGA cohort.

Accession codes

Our mRNA expression data have been deposited into NCBI Gene Expression Omnibus (GEO) (GSE99070). And, the data have been extracted from European Genome-phenome Archive under accession code EGAS00001001563 (*33*), human mRNA gene microarrays (MSKCC; GSE29211) (*34*), mouse mRNA gene expression microarrays (GSE63557) (*42*), and human melanoma mRNA gene expression microarray (GSE78220) (*43*) in NCBI GEO.

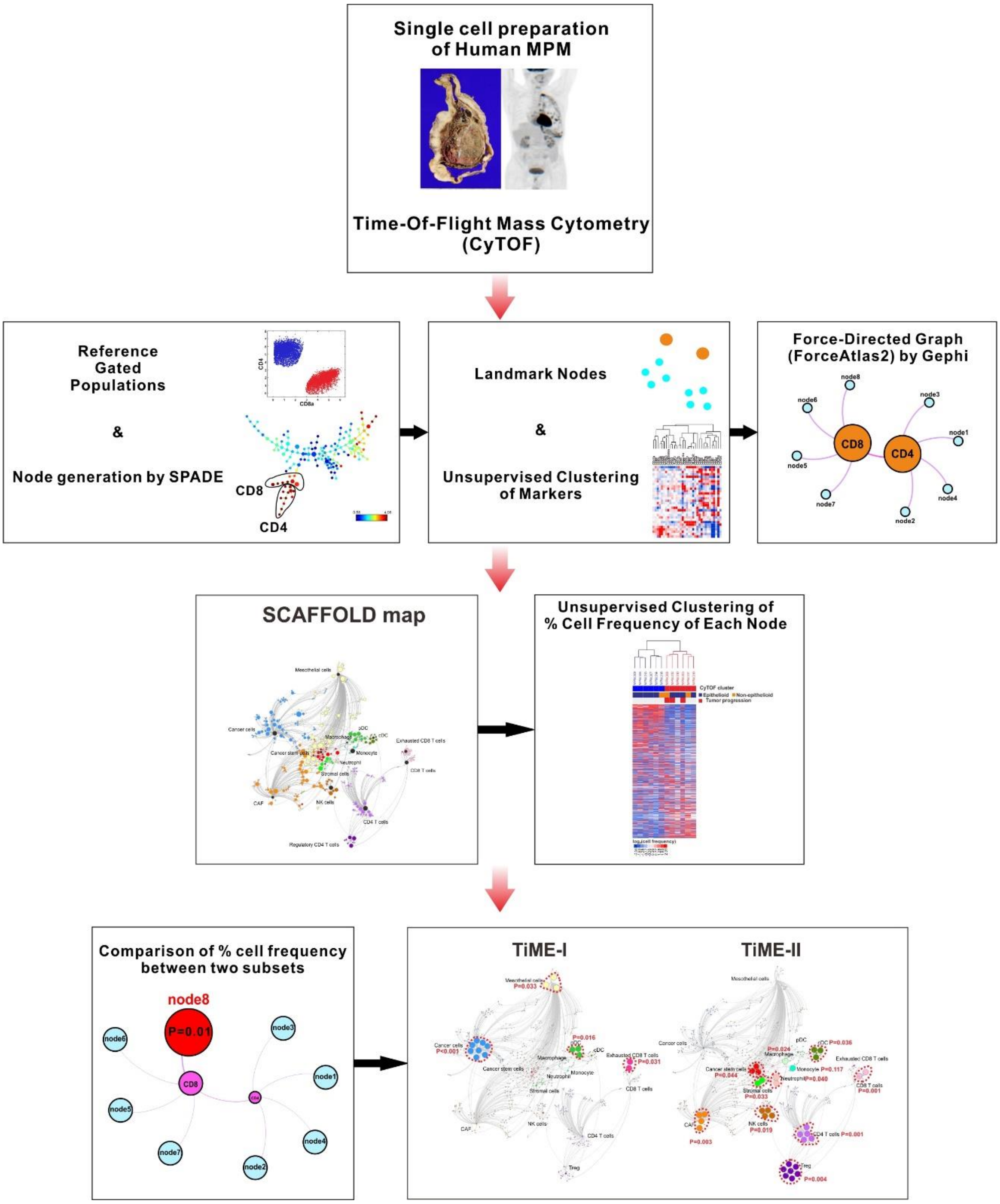

Supplementary Figure 1. Scheme of CyTOF data analysis.

After performing barcoded CyTOF, all nodes and edges are generated through SPADE (Spanning-Tree Progression Analysis of Density-normalized Events) analysis. A SPADE-based SCAFFOLD map is generated.

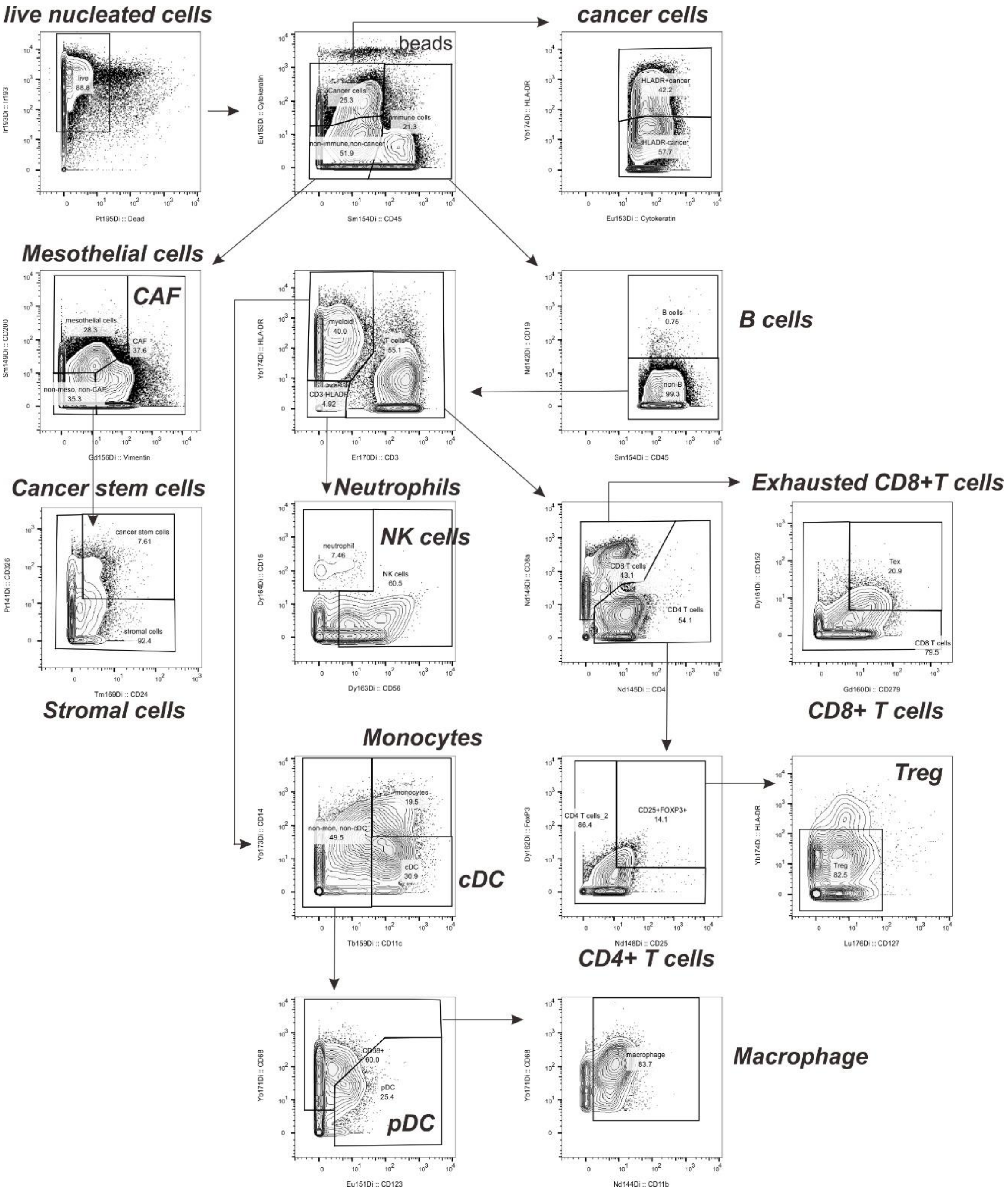

Supplementary Figure 2. Gating of cellular phenotypes in MPM tumors.

To define the phenotypes for representative nodes in the SCAFFOLD map, we manually gated 16 cellular phenotypes by using Flow Jo software. Among them, we utilized 15 cellular phenotypes in SCAFFOLD analyses, as B cell infiltration in MPM was minimal.

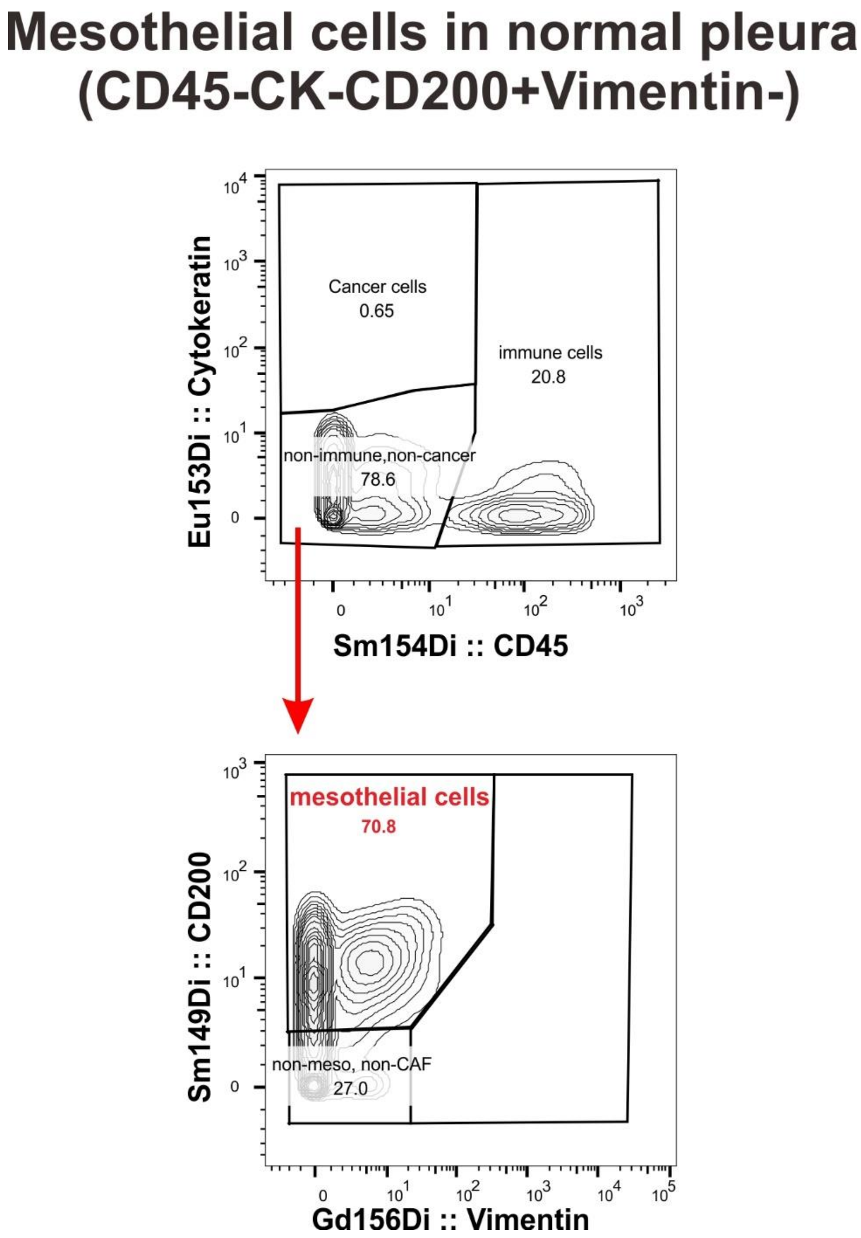

Supplementary Figure 3. Gating of mesothelial cells in normal pleura.

Our definition of mesothelial cells was based on several references (*68-70*) and was confirmed in normal pleural tissue. Normal pleura is mainly composed of mesothelial cells with CD45-PanCK-CD200+ Vim-.

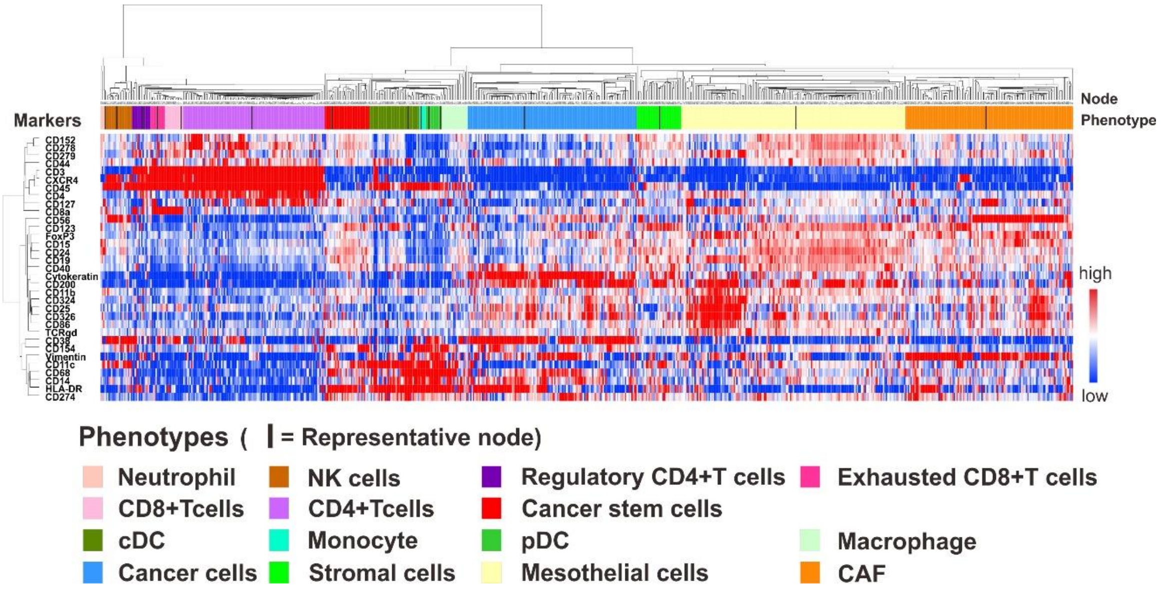

Supplementary Figure 4. Characterizing cell subsets using heatmap analysis of mean protein expression and hierarchical clustering of proteins and populations according to representative nodes.

The heatmap demonstrates characterization of cell populations identified by SPADE (columns) according to mean expression of 33 proteins (rows). Two cell ID markers to detect viable nucleated cells were excluded. Cell populations and proteins were arranged according to average linkage hierarchical clustering. Heat intensity reflects the mean expression of each protein for each cell population. A representative node of each phenotype is displayed as a black bar (▌).

cDC, conventional dendritic cells; NK, natural killer; pDC, plasmacytoid dendritic cells; CAF, cancer-associated fibroblasts.

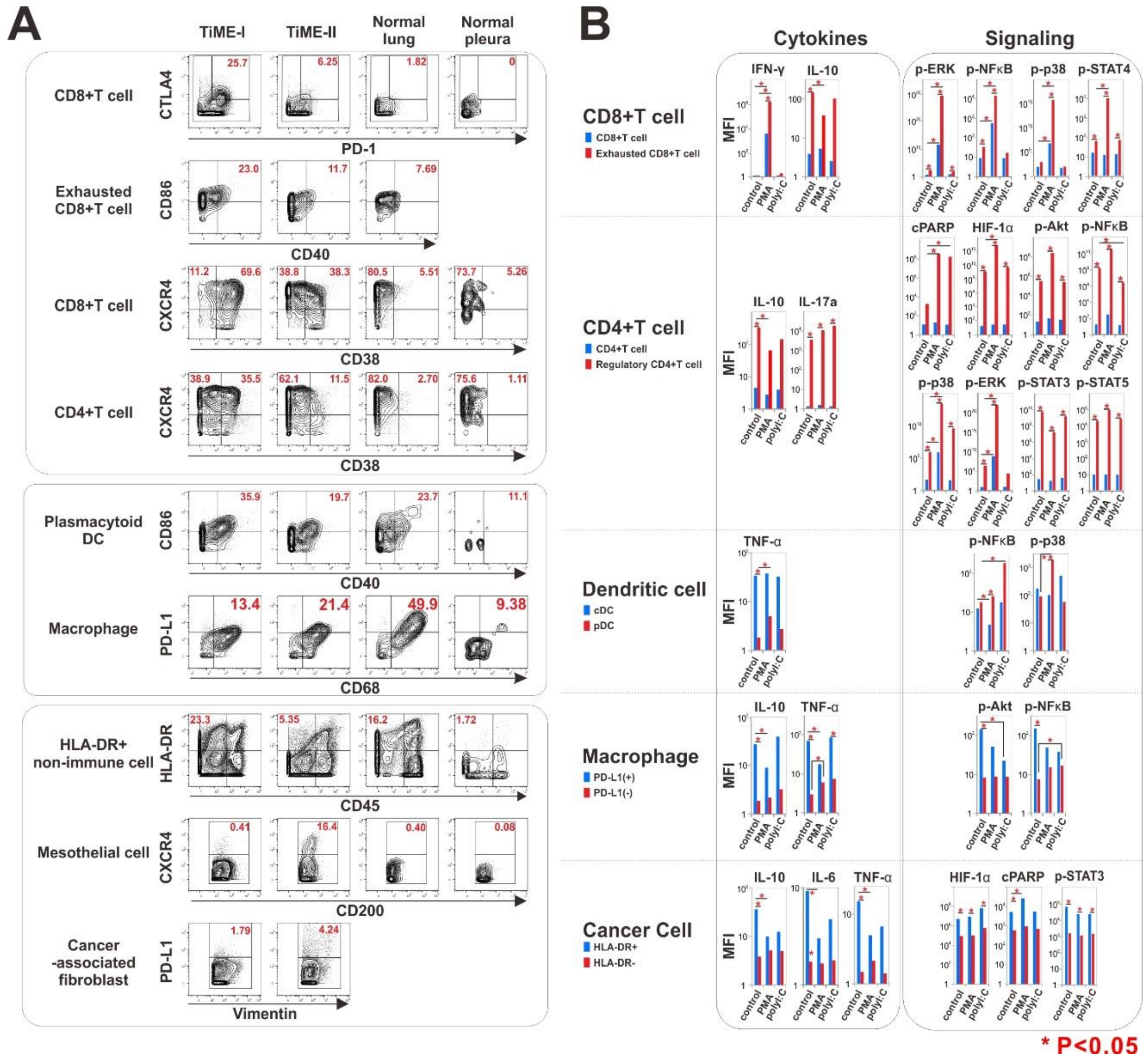

Supplementary Figure 5. Functional evaluation of TiME phenotypes in four patients with epithelioid MPM histology using CyTOF.

(A) Confirmation of CyTOF analysis with 2-D plots using FlowJo V10 software. (B) Functional evaluation of cytokines and phosphosignals in significant phenotypes between TiME-I and TiME-II subsets. TiME-I tumors were enriched for partially-exhausted CD8 T cells that have high capacity to release IFN-γ, activated plasmacytoid dendritic cells (pDC), HLA-DR$^+$ cancer cells, and decreased numbers of IL-10- and IL-17-expressing regulatory CD4 T cells (Tregs) and PD-L1$^+$ tumor-associated macrophages. Mean values were shown.

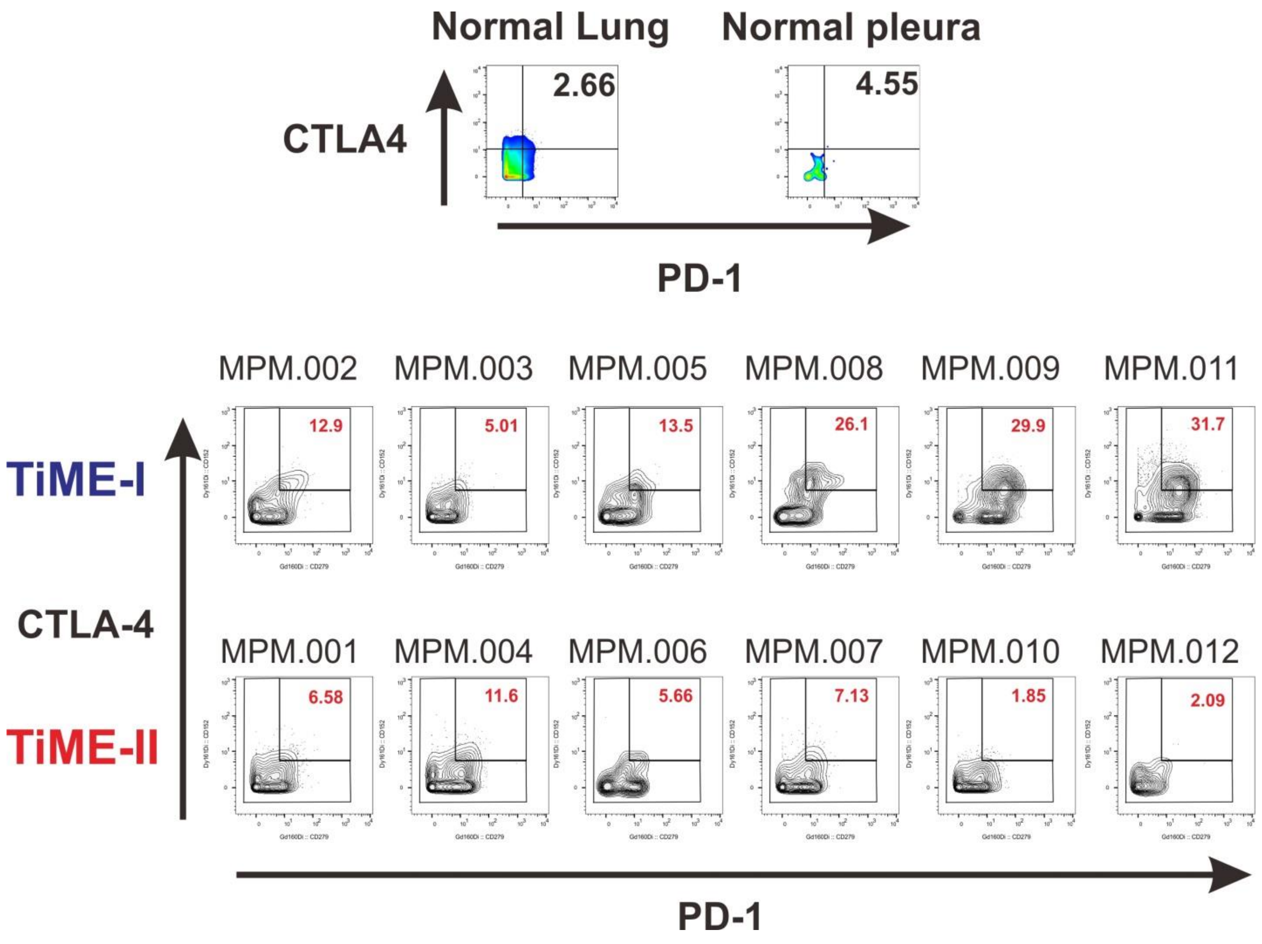

Supplementary Figure 6. Proportion of partially exhausted CD8+ T cells in each patient.

Partially exhausted or dysfunctional T cells are defined as CD8+ T cells that express at least two co-inhibitory molecules such as PD-1 and CTLA-4. In the TiME-I subset, the proportion of partially exhausted T cells exceeded 10% in five out of six patients. However, in the TiME-II subset, five of six patients had less than 10% of exhausted CD8+ T cells. Normal lung and pleura had less than 5% of exhausted CD8+ T cells.

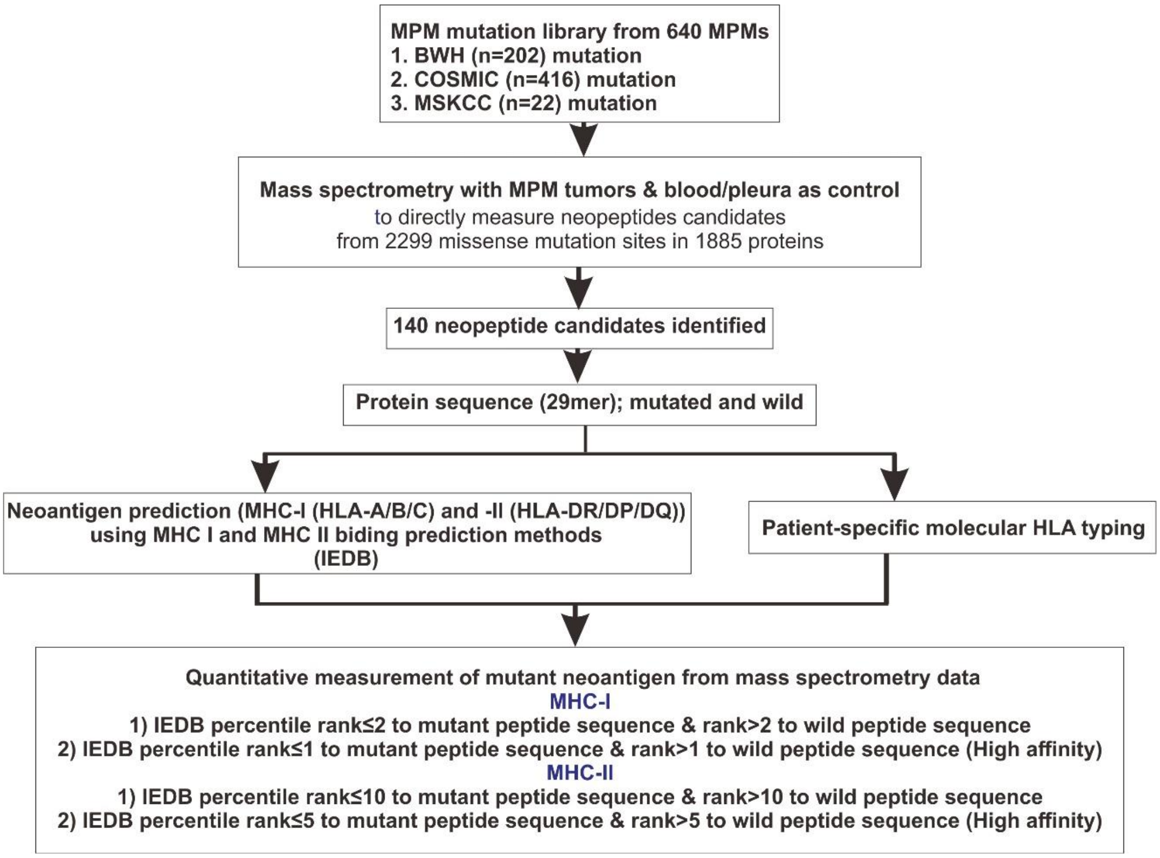

Supplementary Figure 7. Schema for direct identification of mutated neopeptides using mass spectrometry.

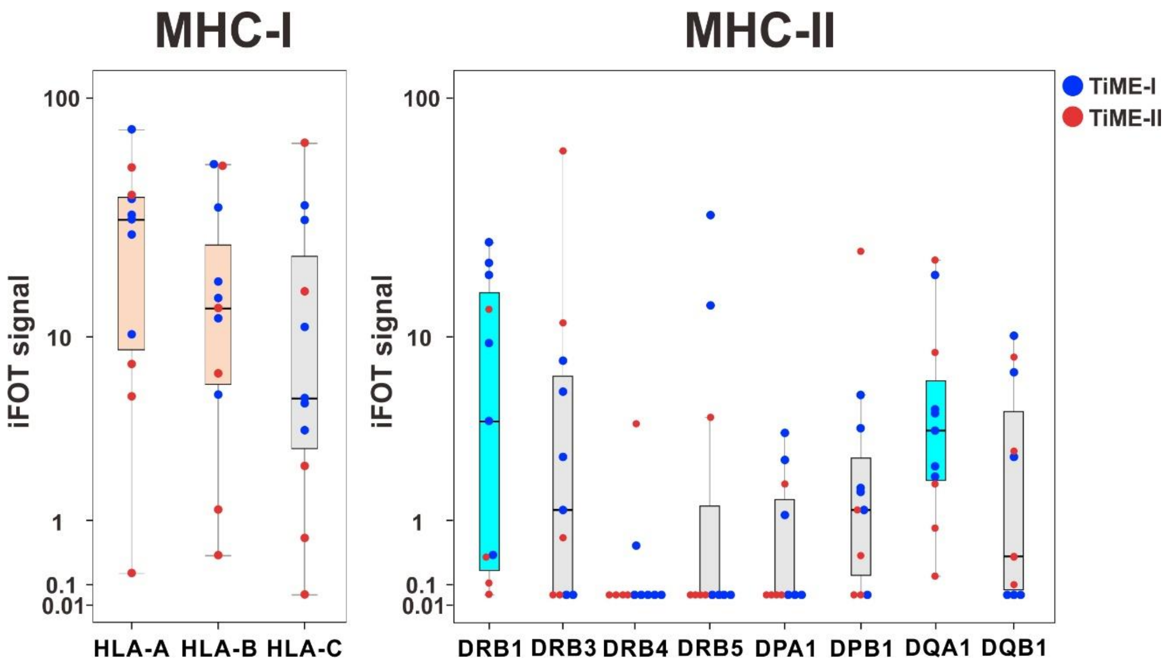

Supplementary Figure 8. Expression of MHC-I and -II proteins in TiME-I and TiME-II tumors.

For MHC-I, the median values of HLA-A and HLA-B are higher than that of HLA-C. For MHC-II, the median values of HLA-DRB1 and HLA-DQA1 are higher than those of other MHC-II molecules. Box plots demonstrate median, 25th percentile (lower), and 75th percentile (upper).

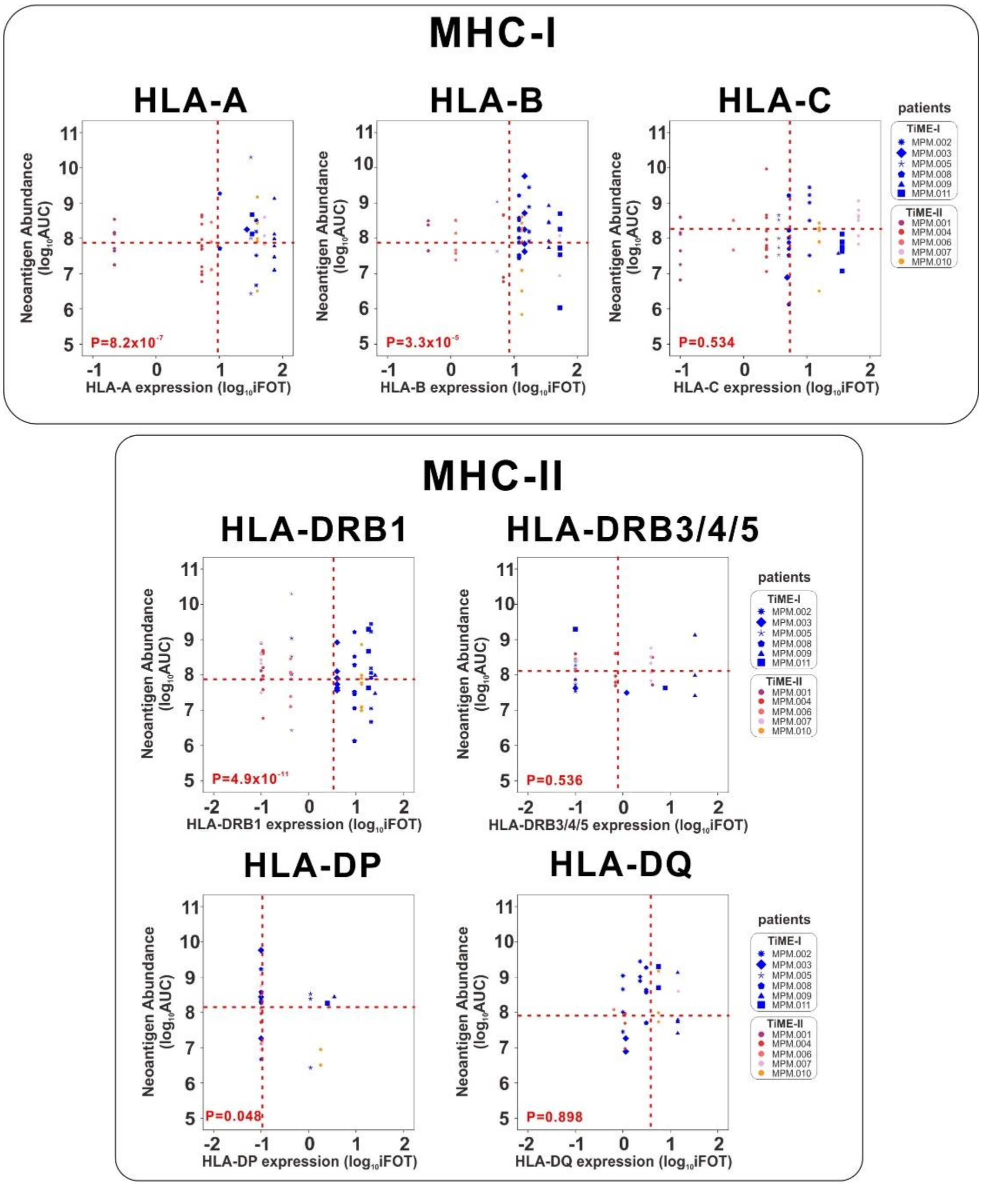

Supplementary Figure 9. Correlation of neoantigen abundance and their corresponding MHC-I and MHC-II proteins between TiME-I and TiME-II tumors.

To evaluate the distribution of neoantigen abundance and the corresponding MHC molecules between TiME-I and TiME-II tumors, we performed chi-square or Fisher's exact tests in four quadrants that are defined by the median values of each axis. Further comparison was then performed between the right upper quadrant (high neoantigen abundance and high expression of MHC proteins) and the other quadrants. Both higher expression of HLA-A, HLA-B, HLA-DRB1, and HLA-DP and higher neoantigen abundance of their binding peptides were significantly associated with TiME-I subset. In these 2D plots, the vertical dot line is the median value of MHC molecules, and horizontal dot line is the median value of neoantigen abundance.

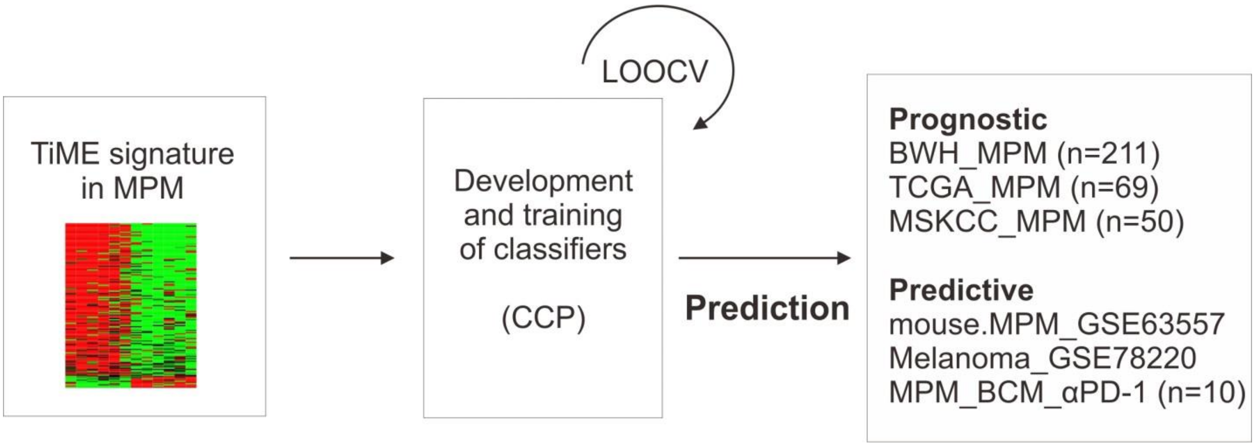

Supplementary Figure 10. Development and validation of predictive classifier using a TiME signature generated from mass spectrometry and mRNA data.

Gene-expression data in the training sets are combined to form a series of classifiers according to the compound covariate predictor (CCP) algorithm and the robustness of the classifier is estimated by the misclassification rate determined during leave-one-out cross-validation (LOOCV) of the training sets. After LOOCV, the sensitivity and specificity of the prediction models are estimated by the fraction of samples correctly predicted.

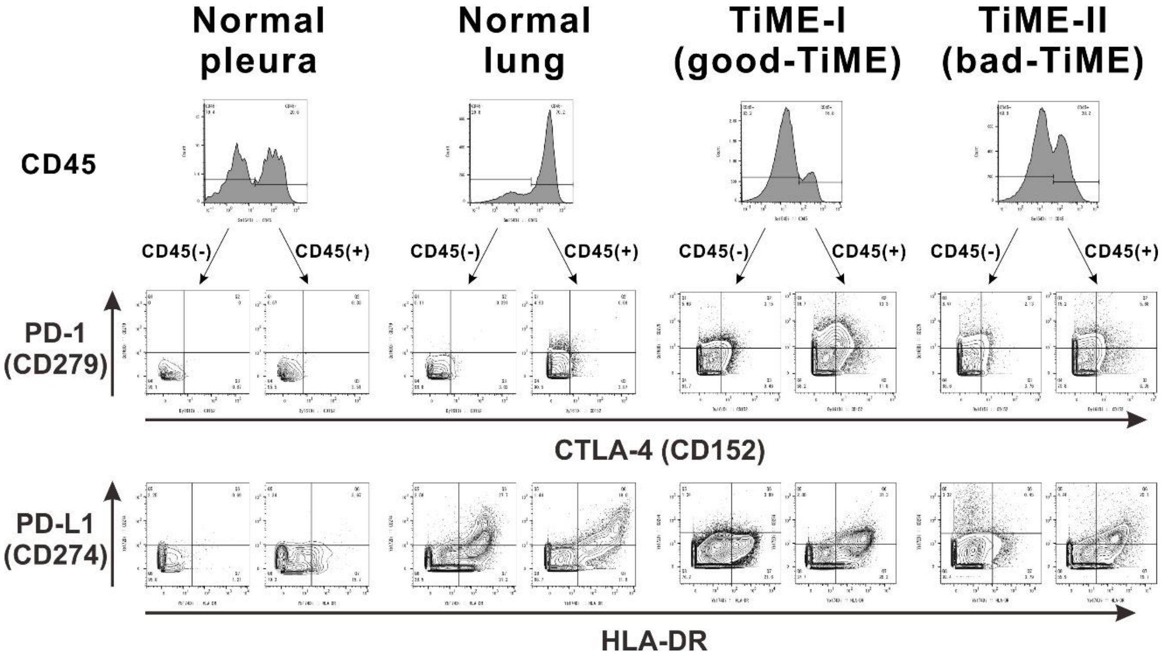

Supplementary Figure 11. Expression of co-inhibitory molecules in MPM.

Representative immune co-inhibitory molecules such as PD-1, CTLA-4, and PD-L1 are elevated in MPM tumors. Normal pleura does not express them. Normal lung has very low expression of PD-1 and CTLA-4 in CD45+ cells, and high expression of PD-L1 on CD45+ cells likely due to its expression on alveolar macrophages. Tumors in TiME-I (good-TiME) subset have high expression of PD-1, CTLA-4, and PD-L1 on immune cells, and low expression of PD-L1 on non-immune cells. In contrast, tumors of the TiME-II (bad-TiME) subset demonstrate lower expression of PD-1 and CTLA-4 on CD45+ cells, and high expression of PD-L1 on both tumor cells and CD45+ cells.

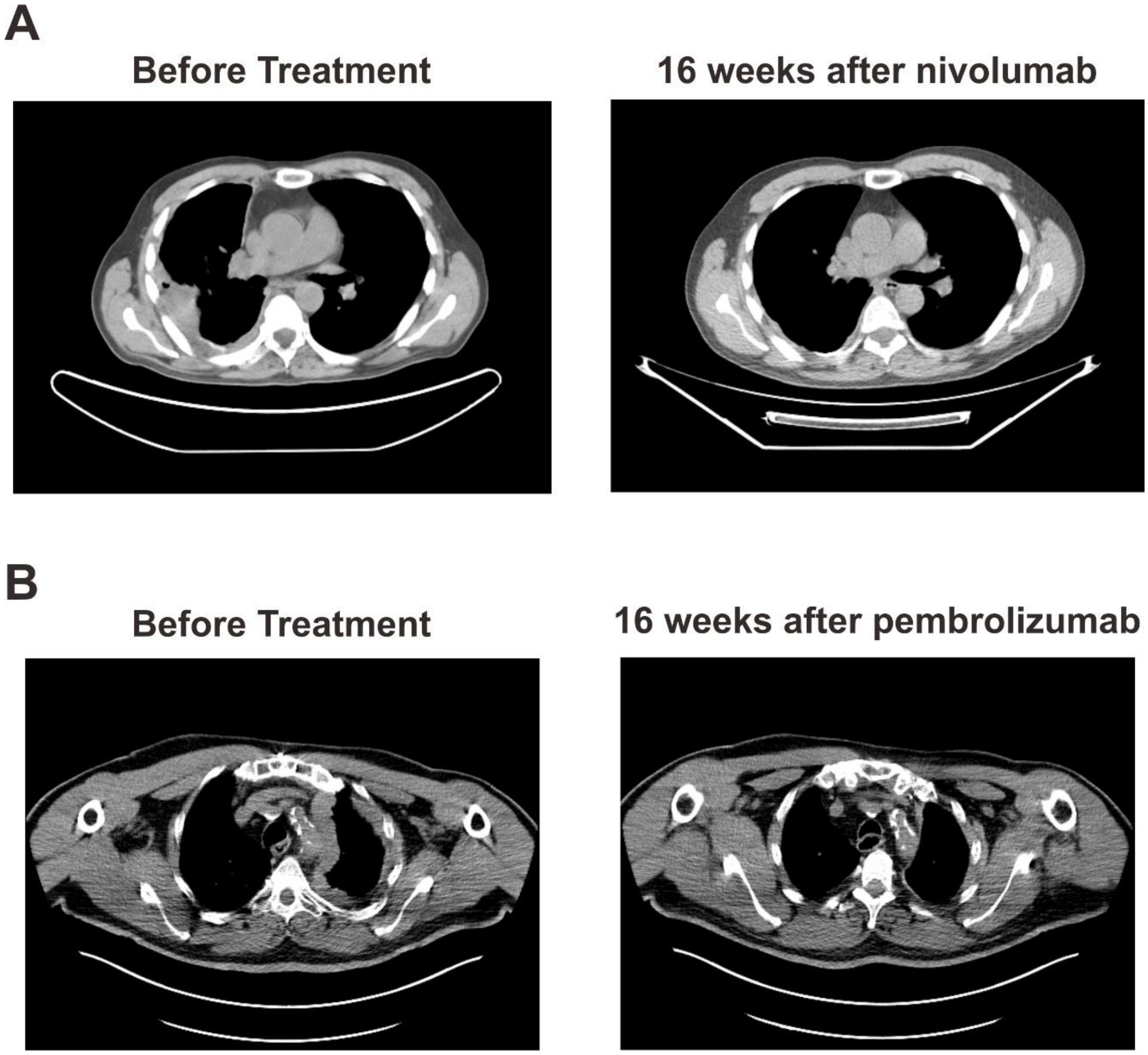

Supplementary Figure 12. Chest CT findings of complete responders to anti-PD-1 monoclonal antibody therapy in MPM.

(A) Complete responder at 16 weeks after nivolumab treatment. (B) Complete responder at 16 weeks after pembrolizumab treatment.

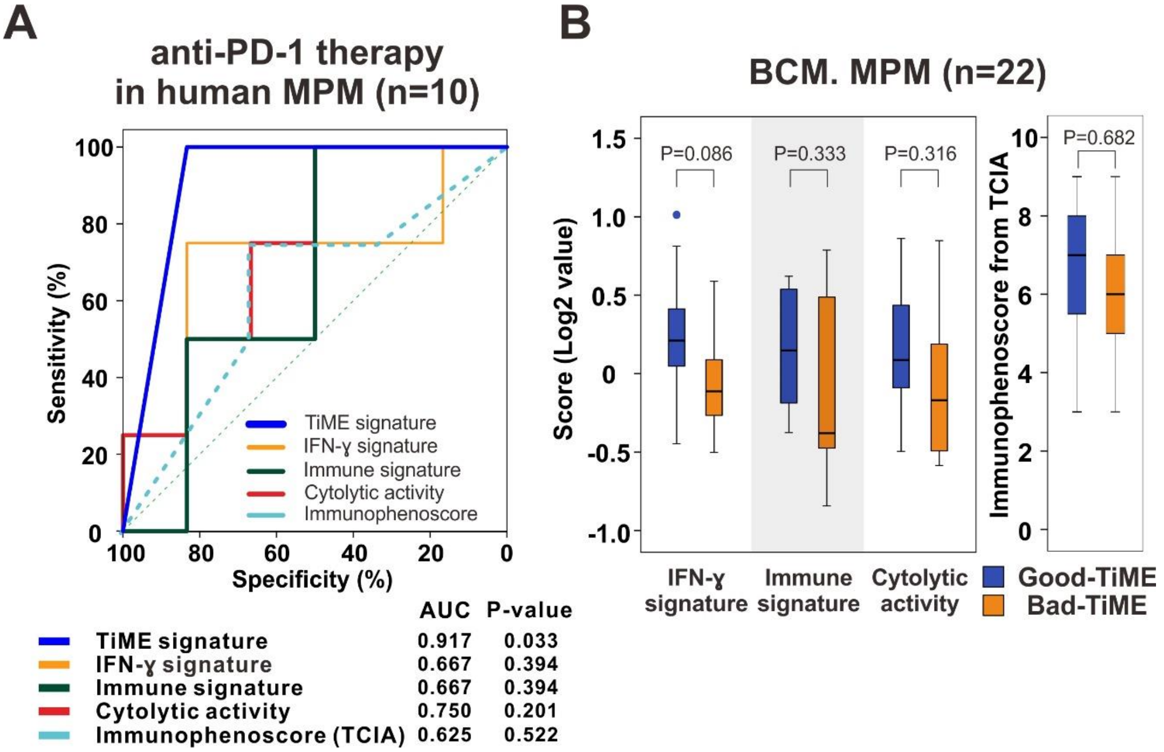

Supplementary Figure 13. Performance of the TiME signature to predict response to anti-PD-1 therapy in patients with MPM is compared to other previously published immune signatures.

(A) Compared to previously reported signatures that predict response to immune checkpoint inhibitors in other malignancies, we tested the performance of an immune signature (CD2, CD247, CD3E, GZMH, GZMK, NKG7 and PRF1), the IFN-γ signature (CIITA, GBP4, GBP5, IRF1, IRF2, and JAK2), cytolytic activity signature (GZMA and PRF1), and the immunophenoscore in 10 MPM patients treated PD-1 blockade. The TiME signature was the strongest predictor of response to anti-PD-1 therapy. In these analyses we considered the average of the expression value of all the genes in the corresponding signature. (B) The IFN-γ signature score had a higher tendency in good-TiME than bad-TiME.

A

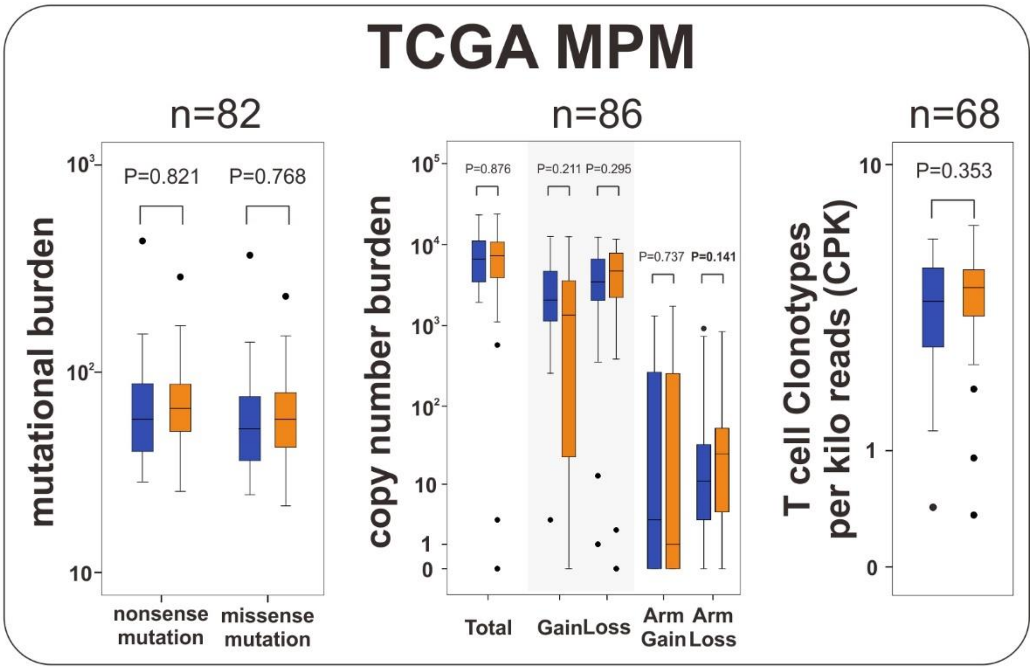

B

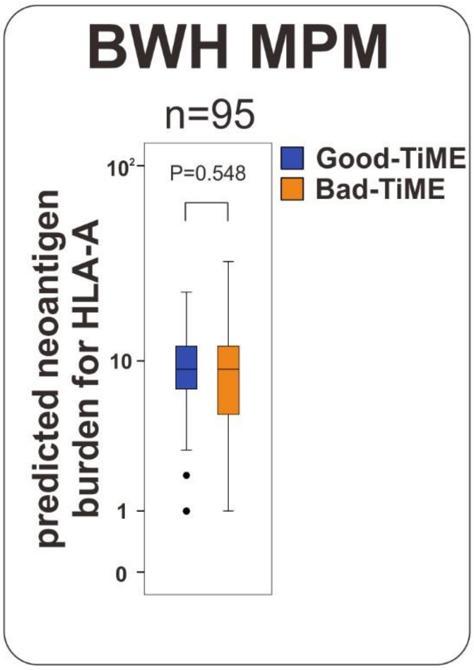

Supplementary Figure 14. Immunogenomic Determinants between good-TiME and bad-TiME.

(A) Comparison of mutational burden, copy number burden, and T cell clonality in TCGA cohort between good-TiME and bad-TiME. (B) Comparison of predicted neoantigen burden for HLA-A in BWH cohort between two subsets.

Supplementary Table 1. Clinicopathologic Characteristics of MPM Patients.

Variables		BCM CyTOF cohort	BWH cohort	TCGA cohort	MSKCC cohort
**Number of patients**		**12**	**211**	**69**	**50**
**Sex**	Male	10 (83.3%)	176 (83.4%)	54 (78.3 %)	33 (66%)
	Female	2 (16.7%)	35 (16.6%)	15 (21.7 %)	17 (34%)
**Age (y), median (range)**		71.1 (55-79)	65.4 (18-86)	64 (28-81)	64 (33-78)
**History of asbestos exposure**		4 (33.3%)	145 (68.7%)	45 (65.2%)	25 (52%)
**Preoperative treatment**		0	35 (16.6%)	-	-
**Pathologic T status**	0	-	1 (0.5%)	-	-
	1	-	10 (4.7%)	12 (17.4%)	6 (12%)
	2	-	48 (22.7%)	22 (31.9%)	17 (34%)
	3	-	88 (41.7%)	27 (39.1%)	15 (30%)
	4	-	37 (17.5%)	6 (8.7%)	10 (20%)
	X	-	2 (0.9%)	2 (2.9%)	
	NA	-	25 (11.8%)	-	2 (4%)
**Pathologic N status**	0	3 (25.0%)	74 (35.1%)	37 (53.6%)	23 (46%)
	1	4 (33.3%)	37 (17.5%)	10 (14.5%)	-
	2	1 (8.4%)	63 (29.9%)	17 (24.6%)	14 (28%)
	3	0	2 (0.9%)	2 (2.9%)	-
	X	4 (33.3%)	10 (4.7%)	3 (4.3%)	11 (22%)
	NA	-	25 (11.8%)	-	2 (4%)
**Histology**	Epithelioid	9 (75.0%)	141 (66.8%)	48 (69.6%)	36 (72%)
	Biphasic	3 (25.0%)	65 (29.4%)	16 (23.2%)	10 (20%)
	Sarcomatoid	-	7 (3.3%)	2 (2.9%)	4 (8%)
	NOS	-	1 (0.5%)	3 (4.3%)	-
**Pathologic TNM stage**	I	-	-	9 (13%)	7 (14%)
	II	-	-	13 (18.8%)	11 (22%)
	III	-	-	38 (55.1%)	19 (38%)
	IV	-	-	9 (13%)	11 (22%)
	NA	-	-	-	2 (4%)
**Follow-up (mo.), median**		6.2	46.3	51.8	126.2

BCM, Baylor College of Medicine; BWH, Brigham Women's Hospital; TCGA, The Cancer Genome Atlas; MSKCC, Memorial Sloan Kettering Cancer Center; NOS, not otherwise specified.

* The pathologic TNM stage was determined according to 7th edition of the American Joint Committee on Cancer staging manual. The staging system in BWH and TCGA cohorts has been combined with that in the 6th edition.

Supplementary Table 2. CyTOF panels.

Surface Marker		Cytokine Marker		Phospho-signal Marker	
**Metal label**	**Specificity**	**Metal label**	**Specificity**	**Metal label**	**Specificity**
**139La**		**139La**	CD56		
**141Pr**	CD326(EpCAM)	**141Pr**	CD326(EpCAM)	**141Pr**	CD235
**142Nd**	CD19	**142Nd**	CD19	**142Nd**	CD19
**143Nd**		**143Nd**	IL-5	**143Nd**	cPARP
**144Nd**	CD11b	**144Nd**	CD11b	**144Nd**	CD11b
**145Nd**	CD4	**145Nd**	CD4	**145Nd**	CD4
**146Nd**	CD8a	**146Nd**	CD8a	**146Nd**	CD8a
**147Sm**	CD278(ICOS)	**147Sm**	CD278(ICOS)	**147Sm**	p-Stat5(Y694)
**148Nd**	CD25	**148Nd**	CD34	**148Nd**	p-Stat4(Y693)
**149Sm**	CD200	**149Sm**	CD200	**149Sm**	pNFKB
**150Nd**	CD86	**150Nd**	CD86	**150Nd**	CD86
**151Eu**	CD123	**151Eu**	CD123	**151Eu**	CD123
**152Sm**	TCRγδ	**152Sm**	TCRγδ	**152Sm**	p-AKT 152Sm
**153Eu**	Cytokeratin(pan)	**153Eu**	Cytokeratin(pan)	**153Eu**	Cytokeratin(pan)
**154Sm**	CD45	**154Sm**	CD45	**154Sm**	CD45
**155Gd**		**155Gd**	CD25	**155Gd**	
**156Gd**	Vimentin	**156Gd**	IL6	**156Gd**	P-p38(180/182)
**158Gd**	CD324 (E-cadherin)	**158Gd**	IFN-γ	**158Gd**	p-STAT3(Y705)
**159Tb**	CD11c	**159Tb**	CD11c	**159Tb**	CD11c
**160Gd**	CD279(PD1)	**160Gd**	CD279(PD1)	**160Gd**	CD279(PD1)
**161Dy**	CD152(CTLA-4)	**161Dy**	CD152(CTLA-4)	**161Dy**	CD152(CTLA-4)
**162Dy**	FOXP3	**162Dy**	FOXP3	**162Dy**	FOXP3
**163Dy**	CD56	**163Dy**	CD15	**163Dy**	CD56
**164Dy**	CD15	**164Dy**	IL-17A	**164Dy**	P-mTOR
**165Ho**	CD40	**165Ho**	CD40	**165Ho**	HIF-1a
**166Er**	CD44	**166Er**	IL-10	**166Er**	CD44
**167Er**	CD38	**167Er**	CD38	**167Er**	p-ERK1/2
**168Er**	CD154 (CD40L)	**168Er**	CD154 (CD40L)	**168Er**	pStat6(Y641)
**169Tm**	CD24	**169Tm**	CXCR4	**169Tm**	CD25
**170Er**	CD3	**170Er**	CD3	**170Er**	CD3
**171Yb**	CD68	**171Yb**	CD68	**171Yb**	CD68
**172Yb**	CD274(PD-L1)	**172Yb**	CD274(PD-L1)	**172Yb**	CD274(PD-L1)
**173Yb**	CD14	**173Yb**	CD14	**173Yb**	CD14
**174Yb**	HLA-DR	**174Yb**	HLA-DR	**174Yb**	HLA-DR
**175Lu**	CXCR4	**175Lu**	TNF-α	**175Lu**	p-S6
**176Yb**	CD127	**176Yb**	CD127	**176Yb**	CD127
**191Ir**	Ir	**191Ir**	Ir	**191Ir**	Ir
**195Pt**	viability	**195Pt**	viability	**195Pt**	viability
**Total**	35		38		36

Supplementary Table 3. Library of peptides with missense mutation in MPM.

Table is provided in Other Supplementary Material as an Excel file

(Table S3 in Supplementary.Tables.xls).

Supplementary Table 4. Directly identified peptides with mutation by Mass Spectrometry.

Mutated peptides	Mutated sequence	Wild sequence
A2ML1_G922R	VLVEKTHSSLLCPKGKVASESVSLELPVD	VLVEKTHSSLLCPKRKVASESVSLELPVD
AASS_V28I	VSLSKGLHHKAVLAVRREDVNAWERRAPL	VSLSKGLHHKAVLAIRREDVNAWERRAPL
ACTC1_A137V	QIMFETFNVPAMYVAIQAVLSLYASGRTT	QIMFETFNVPAMYVVIQAVLSLYASGRTT
ACTL8_S261R	ELTPMQRVAPEMFFSPQVFEQPGPSIPRA	ELTPMQRVAPEMFFRPQVFEQPGPSIPRA
ADAM30_V624I	TSCGEGRVCFKKNCVNSSVLQFDCLPEKC	TSCGEGRVCFKKNCINSSVLQFDCLPEKC
ADAMTSL1_D465N	TCGQGLRYRVVLCIDHRGMHTGGCSPKTK	TCGQGLRYRVVLCINHRGMHTGGCSPKTK
AHNAK_D1670E	PDVDLHLKGPKVKGDMDVSVPKVEGEMKV	PDVDLHLKGPKVKGEMDVSVPKVEGEMKV
AHNAK2_P1594L	QMPSFKMPKVDLKGPQIDVKGPKLDLKGP	QMPSFKMPKVDLKGLQIDVKGPKLDLKGP
ANK1_T1802M	SQEYEKVLVSVSEHTWTEQPEAESSQADR	SQEYEKVLVSVSEHMWTEQPEAESSQADR
ARHGAP29_R162Q	TNFLMGDVGNDSLLRLPVSRETKSFENVS	TNFLMGDVGNDSLLQLPVSRETKSFENVS
ASAP1_A545T	TPSSDMTVRKEYITAKYVDHRFSRKTCST	TPSSDMTVRKEYITTKYVDHRFSRKTCST
AZI2_E177Q	LSCDLKIHGLEQELELMRKECSDLKIELQ	LSCDLKIHGLEQELQLMRKECSDLKIELQ
B3GALT1_I84S	PNKCEKNIPFLVILISTTHKEFDARQAIR	PNKCEKNIPFLVILSSTTHKEFDARQAIR
BAP1_G686D	DEFICTFISMLAQEGMLANLVEQNISVRR	DEFICTFISMLAQEDMLANLVEQNISVRR
BAP1_N645K	ELLALLKCVEAEIANYEACLKEEVEKRKK	ELLALLKCVEAEIAKYEACLKEEVEKRKK
BHLHB9_E95Q	LGKAMGDFTPKAGNESTSSTCKNEAGTDA	LGKAMGDFTPKAGNQSTSSTCKNEAGTDA
BMS1_R690G	AEAFLRQQQAAPNLRKLIYGTVTEDNEEE	AEAFLRQQQAAPNLGKLIYGTVTEDNEEE
BRD8_P580L	GDETPLTNVKTEASPESMLSPSHGSNPIE	GDETPLTNVKTEASLESMLSPSHGSNPIE
BTBD19_H45L	VCFVVGQERQEVFAHRCLLACRCNFFQRL	VCFVVGQERQEVFALRCLLACRCNFFQRL
C10orf76_M1V	MAQVEKRGGLLRKSS	VAQVEKRGGLLRKSS
CALR_A72G	GDEEKDKGLQTSQDARFYALSASFEPFSN	GDEEKDKGLQTSQDGRFYALSASFEPFSN
CASP14_E33K	LALILCVTKAREGSEEDLDALEHMFRQLR	LALILCVTKAREGSKEDLDALEHMFRQLR
CCDC85A_H258N	GSPEHSKHRSASPEHPQKPRACGTPDRPK	GSPEHSKHRSASPENPQKPRACGTPDRPK
CELSR3_K3115R	SQECMDAAPGRLEPKDRGSTLPRRQPPRD	SQECMDAAPGRLEPRDRGSTLPRRQPPRD
CFAP45_E465K	RAQREQIEKERLEEEKKATGRLQHANELR	RAQREQIEKERLEEKKKATGRLQHANELR
CKAP2L_N380K	SCVLQKSKAISQRPNLTVGRFNSAIPSTP	SCVLQKSKAISQRPKLTVGRFNSAIPSTP
CLEC16A_V760M	WGVVKFAGLLQDMQVTGVEDDSRALNITI	WGVVKFAGLLQDMQMTGVEDDSRALNITI
COL7A1_R1388C	GPPGPRGPLGDPGPRGPPGLPGTAMKGDK	GPPGPRGPLGDPGPCGPPGLPGTAMKGDK
CSF2RA_R297K	FPSSEPRAKHSVKIRAADVRILNWSSWSE	FPSSEPRAKHSVKIKAADVRILNWSSWSE
CSNK1A1L_E10K	MTNNSGSKAELVVGGKYKLVRKIG	MTNNSGSKAKLVVGGKYKLVRKIG
CSPP1_S1121A	SSRPNVAPDGLSLKSISSVNVDELRVRNE	SSRPNVAPDGLSLKAISSVNVDELRVRNE
CTNNA2_V134G	RGTMVRAARALLSAVTRLLILADMADVMR	RGTMVRAARALLSAGTRLLILADMADVMR
CWC25_R90C	GPGGMVNRDEYLLGRPIDKYVFEKMEEKE	GPGGMVNRDEYLLGCPIDKYVFEKMEEKE
CYP4A11_A336S	GHDTTASGISWILYALATHPKHQERCREE	GHDTTASGISWILYSLATHPKHQERCREE
DCX_E302Q	FEQVLTDITEAIKLETGVVKKLYTLDGKQ	FEQVLTDITEAIKLQTGVVKKLYTLDGKQ
DENND4B_L307M	RALGLLSAVERGRALGGRAVRSRRAIAVL	RALGLLSAVERGRAMGGRAVRSRRAIAVL
DHRS4_T102M	CHVGKAEDRERLVATAVKLHGGIDILVSN	CHVGKAEDRERLVAMAVKLHGGIDILVSN
DNAAF2_V200M	KTLKAKYKGTPEAAVLRTPLPGVIPARPD	KTLKAKYKGTPEAAMLRTPLPGVIPARPD

DNAH6_R364Q	VIRLAEVTERLGEFRNEAKYVVRRACRFA	VIRLAEVTERLGEFQNEAKYVVRRACRFA
DNM1_I248F	VVNRSQKDIDGKKDITAALAAERKFFLSH	VVNRSQKDIDGKKDFTAALAAERKFFLSH
DOCK8_R436L	TDVDSVVGRSSVGERRTLAQSRRLSERAL	TDVDSVVGRSSVGELRTLAQSRRLSERAL
EFCAB7_M168I	ADGKFDYIKFCKLYMTTNEQCLKTTLEKL	ADGKFDYIKFCKLYITTNEQCLKTTLEKL
EHMT1_L742V	DSEKPKKLRFHPKQLYFSARQGELQKVLL	DSEKPKKLRFHPKQVYFSARQGELQKVLL
FAM129B_L249P	QILSNLVMEELGPELKAELGPRLKGKPQE	QILSNLVMEELGPEPKAELGPRLKGKPQE
FAM179A_L133F	RDTIQIKDKLKKRRLSEGLAASSRASLDP	RDTIQIKDKLKKRRFSEGLAASSRASLDP
FAM86B2_E82K	KYAWCFLSELIKKHEAVHTEPLDKLYEVL	KYAWCFLSELIKKHKAVHTEPLDKLYEVL
FANCF_L8V	MESLLQHLDRFSELLAVSSTTY	MESLLQHVDRFSELLAVSSTTY
FLII_M1196V	CSDFCQDDLADDDIMLLDNGQEVYMWVGT	CSDFCQDDLADDDIVLLDNGQEVYMWVGT
FLT1_V155A	EIPEIIHMTEGRELVIPCRVTSPNITVTL	EIPEIIHMTEGRELAIPCRVTSPNITVTL
FLT4_R477Q	WTPCKMFAQRSLRRRQQQDLMPQCRDWRA	WTPCKMFAQRSLRRQQQQDLMPQCRDWRA
GGNBP2_E632K	DSGKGAKSLVELLDESECTSDEEIFISQD	DSGKGAKSLVELLDKSECTSDEEIFISQD
GIMAP1_S41R	TRRLILVGRTGAGKSATGNSILGQRRFFS	TRRLILVGRTGAGKRATGNSILGQRRFFS
GPATCH2_I223M	VKKRKLKIIRQGPKIQDEGVVLESEETNQ	VKKRKLKIIRQGPKMQDEGVVLESEETNQ
GPR12_T116M	VFAYLLQSEATKLVTIGLIVASFSASVCS	VFAYLLQSEATKLVMIGLIVASFSASVCS
GRB14_P316S	TNYGFCFKPNKAGGPRDLKMLCAEEEQSR	TNYGFCFKPNKAGGSRDLKMLCAEEEQSR
GRK6_P581S	ATARKSSPPASSPQPEAPTSSWR	ATARKSSPPASSPQSEAPTSSWR
HERC1_I1657L	QILVLLSGMEEKGSISLAGSRLSSGFQSS	QILVLLSGMEEKGSLSLAGSRLSSGFQSS
HERC2_R1211C	TGQNCRNNEEVTLIRKADLENHNKDGGFW	TGQNCRNNEEVTLICKADLENHNKDGGFW
HOXD8_Q184P	GEDPDHLNQSSSPSQMFPWMRPQAAPGRR	GEDPDHLNQSSSPSPMFPWMRPQAAPGRR
IGLV3-1_D48E	GQTASITCSGDKLGDKYACWYQQKPGQSP	GQTASITCSGDKLGEKYACWYQQKPGQSP
IL23R_I80S	FYKNGIKERFQITRINKTTARLWYKNFLE	FYKNGIKERFQITRSNKTTARLWYKNFLE
IMMT_D492H	EMRTQLRRQAAAHTDHLRDVLRVQEQELK	EMRTQLRRQAAAHTHHLRDVLRVQEQELK
INHBC_C316S	LKANTAAGTTGGGSCCVPTARRPLSLLYY	LKANTAAGTTGGGSSCVPTARRPLSLLYY
INTS3_A991P	YEDSSTKPPKSRRKAALSSPRSRKNATQP	YEDSSTKPPKSRRKPALSSPRSRKNATQP
IRF8_D153N	GRSEIDELIKEPSVDDYMGMIKRSPSPPE	GRSEIDELIKEPSVNDYMGMIKRSPSPPE
ITPR2_L2065P	IMESRHDSENAERILFNMRPRELVDVMKN	IMESRHDSENAERIPFNMRPRELVDVMKN
KAT2A_E831K	CASALEKFFYFKLKEGGLIDK	CASALEKFFYFKLKKGGLIDK
KCNA5_V588I	DSARRGSCPLEKCNVKAKSNVDLRRSLYA	DSARRGSCPLEKCNIKAKSNVDLRRSLYA
KCND3_P31A	IGWMPVANCPMPLAPADKNKRQDELIVLN	IGWMPVANCPMPLAAADKNKRQDELIVLN
KCNJ3_E449K	MKLQRISSVPGNSEEKLVSKTTKMLSDPM	MKLQRISSVPGNSEKKLVSKTTKMLSDPM
KIAA0430_L185V	GIASDFPSMCLESNLSSCKHLPCCGKLHF	GIASDFPSMCLESNVSSCKHLPCCGKLHF
KIF21B_M810L	FQIRALESQKRQQEMVLRRKTQEVSALRR	FQIRALESQKRQQELVLRRKTQEVSALRR
KLHL5_D570H	VAVLEGPMYAVGGHDGWSYLNTVERWDPQ	VAVLEGPMYAVGGHHGWSYLNTVERWDPQ
KLKB1_M353V	DCKEEKCKCFLRLSMDGSPTRIAYGTQGS	DCKEEKCKCFLRLSVDGSPTRIAYGTQGS
KRAS_G13S	MTEYKLVVVGAGGVGKSALTIQLIQNHF	MTEYKLVVVGAGSVGKSALTIQLIQNHF
KRT19_E210D	ARTDLEMQIEGLKEELAYLKKNHEEEIST	ARTDLEMQIEGLKEDLAYLKKNHEEEIST
KRT19_V296D	VAGHTEQLQMSRSEVTDLRRTLQGLEIEL	VAGHTEQLQMSRSEDTDLRRTLQGLEIEL
KRT37_A282V	DLNRVLGEMRAQYEAMVETNHQDVEQWFQ	DLNRVLGEMRAQYEVMVETNHQDVEQWFQ
KRT71_S457R	MSGEFPSPVSISIISSTSGGSVYGFRPSM	MSGEFPSPVSISIIRSTSGGSVYGFRPSM

LILRB5_L65F	GPLETEEYRLDKEGLPWARKRQNPLEPGA	GPLETEEYRLDKEGFPWARKRQNPLEPGA
LIX1_A22G	LRHIIAQVLPHRDPALVFKDLNVVSMLQE	LRHIIAQVLPHRDPGLVFKDLNVVSMLQE
LMOD1_A165T	EEKIIRGIDKGRVRAAVDKKEAGKDGRGE	EEKIIRGIDKGRVRTAVDKKEAGKDGRGE
LRRC3_V223M	VTMFGWFAMVIAYVVYYVRHNQEDARRHL	VTMFGWFAMVIAYVMYYVRHNQEDARRHL
MAP1A_V2474E	SIDDRDLSTEEVRLVGRGGRRRVGGPGTT	SIDDRDLSTEEVRLEGRGGRRRVGGPGTT
MAPK1_I347L	MELDDLPKEKLKELIFEETARFQPGYRS	MELDDLPKEKLKELLFEETARFQPGYRS
MDC1_E1872Q	TPKPGKRKRDQAEEEPNRIPSRSLRRTKL	TPKPGKRKRDQAEEQPNRIPSRSLRRTKL
MDN1_P2282R	SERGMIDGSTPTITPNPNFRLFLSMDPVH	SERGMIDGSTPTITRNPNFRLFLSMDPVH
MEIS1_T100P	LLALIFEKCELATCTPREPGVAGGDVCSS	LLALIFEKCELATCPPREPGVAGGDVCSS
MGA_F2443S	TANERRRRGEMRDLFEKLKITLGLLHSSK	TANERRRRGEMRDLSEKLKITLGLLHSSK
MGAT5_L708H	SFDPKNKHCVFQGDLLLFSCAGAHPRHQR	SFDPKNKHCVFQGDHLLFSCAGAHPRHQR
MMS22L_H209R	FVNQNQIKLFPPSWHLLHLHLDIHWLVLE	FVNQNQIKLFPPSWRLLHLHLDIHWLVLE
MOV10_G853R	KIRYCITKLDRELRGLDDIKDLKVGSVEE	KIRYCITKLDRELRRLDDIKDLKVGSVEE
MPP4_H218Y	NGVSVEGLDPEQVIHILAMSRGTIMFKVV	NGVSVEGLDPEQVIYILAMSRGTIMFKVV
MRPL19_S186R	QEIQVVKLEKRLDDSLLYLRDALPEYSTF	QEIQVVKLEKRLDDRLLYLRDALPEYSTF
MRPS22_R50C	LLQPLPCSFEMGLPRRRFSSEAAESGSPE	LLQPLPCSFEMGLPCRRFSSEAAESGSPE
MTMR4_A134V	RPAKPEDLFAFAYHAWCLGLTEEDQHTHL	RPAKPEDLFAFAYHVWCLGLTEEDQHTHL
MUC2_P1732S	TPTPTPISTTTTVTPTPTPTGTQTPTTTP	TPTPTPISTTTTVTSTPTPTGTQTPTTTP
MUC21_T323A	SSGANTATNSDSSTTSSGASTATNSESST	SSGANTATNSDSSTASSGASTATNSESST
MYO16_H227Y	GNVNEKNDEGVTLLHMACASGYKEVVSLI	GNVNEKNDEGVTLLYMACASGYKEVVSLI
NCOA6_Q1052E	MMQQDPKSVRLPVSQNVHPPRGPLNPDSQ	MMQQDPKSVRLPVSENVHPPRGPLNPDSQ
NDRG1_M315L	LAEAFKYFVQGMGYMPSASMTRLMRSRTA	LAEAFKYFVQGMGYLPSASMTRLMRSRTA
NF2_E166V	DYDPSVHKRGFLAQEELLPKRVINLYQMT	DYDPSVHKRGFLAQVELLPKRVINLYQMT
NLE1_D457N	VKAQKLAMDLPGHADEVYAVDWSPDGQRV	VKAQKLAMDLPGHANEVYAVDWSPDGQRV
NSD1_E129V	SLSPGGPTALAMKQEPSCNNSPELQVKVT	SLSPGGPTALAMKQVPSCNNSPELQVKVT
OFD1_E407D	KEELNQSVNRVKELELELESVKAQSLAIT	KEELNQSVNRVKELDLELESVKAQSLAIT
OR10A7_S87Y	FTLVMVPKMLVDLVSPRKIISFVGCGTQM	FTLVMVPKMLVDLVYPRKIISFVGCGTQM
OR4K14_A126S	EMVLLVSMAYDRYVAICKPLHYMTLMSWQ	EMVLLVSMAYDRYVSICKPLHYMTLMSWQ
PARD3_S529L	NGVDLVGKSQEEVVSLLRSTKMEGTVSLL	NGVDLVGKSQEEVVLLLRSTKMEGTVSLL
PDIA4_P591L	GLVIAKMDATANDVPSDRYKVEGFPTIYF	GLVIAKMDATANDVLSDRYKVEGFPTIYF
PIK3AP1_G313R	KLLTESLKNNIPASGLHLFGINQLEEEDM	KLLTESLKNNIPASRLHLFGINQLEEEDM
PLCE1_V2289M	LLTSESIQTKEEKPVGGLSSSDTMDYRQ	LLTSESIQTKEEKPMGGLSSSDTMDYRQ
PLCL1_P403L	FDPEQKKVAQDMTQPLSHYYINASHNTYL	FDPEQKKVAQDMTQLLSHYYINASHNTYL
POTEF_F536S	EPEINKDGDRELENFMAIEEMKKHRSTHV	EPEINKDGDRELENSMAIEEMKKHRSTHV
PPM1F_G386W	VGLVQSHLTRQQGSGLRVAEELVAAARER	VGLVQSHLTRQQGSWLRVAEELVAAARER
PRDM15_R1298Q	QEDLAEGKHGKAAKRSHKRKQKPEEEAGA	QEDLAEGKHGKAAKQSHKRKQKPEEEAGA
PREX2_V1266G	LEYSDSETQLRRDMVFCQTLVATVCAFSE	LEYSDSETQLRRDMGFCQTLVATVCAFSE
PRKDC_T2618I	PMFVETQASQGTLQTRTQEGSLSARWPVA	PMFVETQASQGTLQIRTQEGSLSARWPVA
PTGR1_G315V	YIIEGFENMPAAFMGMLKGDNLGKTIVKA	YIIEGFENMPAAFMVMLKGDNLGKTIVKA
PTGS1_P269L	VLDGEMYPPSVEEAPVLMHYPRGIPPQSQ	VLDGEMYPPSVEEALVLMHYPRGIPPQSQ
RBP3_V282M	KLRIGESDFFFTVPVSRSLGPLGGGSQTW	KLRIGESDFFFTVPMSRSLGPLGGGSQTW

RGAG4_P547L	RRTGRLGQRQVRRRPPVLFRLTPRQGGHR	RRTGRLGQRQVRRRLPVLFRLTPRQGGHR
SETDB1_M154L	AGSRTPKDQKLREAMAALRKSAQDVQKFM	AGSRTPKDQKLREALAALRKSAQDVQKFM
SLIT1_R1505P	SCPGQGCCQGLRLKRRKFTFECSDGTSFA	SCPGQGCCQGLRLKPRKFTFECSDGTSFA
SMARCAD1_V766I	LGLFNRLKKSINNLVTEKNTEMCNVMMQL	LGLFNRLKKSINNLITEKNTEMCNVMMQL
SNX11_E12D	MGFWCRMSENQEQEEVITVRVQDPRV	MGFWCRMSENQDQEEVITVRVQDPRV
SWAP70_D253N	ISYYVSEDLKDKKGDILLDENCCVESLPD	ISYYVSEDLKDKKGNILLDENCCVESLPD
SYNRG_G431C	LGQPVMGINLVGPVGGAAAQASSGFIPTY	LGQPVMGINLVGPVCGAAAQASSGFIPTY
T_L75V	EMIVTKNGRRMFPVLKVNVSGLDPNAMYS	EMIVTKNGRRMFPVVKVNVSGLDPNAMYS
TANC1_P296L	ESTLPKAESSAGDGPVPYSQGSSSLIMPR	ESTLPKAESSAGDGLVPYSQGSSSLIMPR
TBC1D1_T1029S	QHENLETIVDFIKSTLPNLGLVQMEKTIN	QHENLETIVDFIKSSLPNLGLVQMEKTIN
TGM1_I523V	DDGSFKIVYVEEKAIGTLIVTKAISSNMR	DDGSFKIVYVEEKAVGTLIVTKAISSNMR
TMPRSS15_A146P	SDENVKEELIQGLEANKSSQLVTFHIDLN	SDENVKEELIQGLEPNKSSQLVTFHIDLN
TP53_H179R	QSQHMTEVVRRCPHHERCSDSDGLAPPQH	QSQHMTEVVRRCPHRERCSDSDGLAPPQH
UBQLN4_R407L	NPQLMQNVISAPYMRSMMQTLAQNPDFAA	NPQLMQNVISAPYMLSMMQTLAQNPDFAA
UBR2_L1755S	QEANQTLVGIDWQHL	QEANQTLVGIDWQHS
USP29_L798F	LGALGSDNPGNKNILDAENTRGEAKELTR	LGALGSDNPGNKNIFDAENTRGEAKELTR
UTRN_A1272T	STEVLPEKTDAVNEALESLESVLRHPADN	STEVLPEKTDAVNETLESLESVLRHPADN
VAMP1_P8L	MSAPAQPPAEGTEGTAPGGGPP	MSAPAQPLAEGTEGTAPGGGPP
WFIKKN2_T197A	NTSPPPPETTMHPTTASPETPELDMAAPA	NTSPPPPETTMHPTAASPETPELDMAAPA
ZMYND8_K353R	NNCYLMSKEIPFSVKKTKSIFNSAMQEME	NNCYLMSKEIPFSVRKTKSIFNSAMQEME

Supplementary Table 5. HLA molecular typing of MPM patients.

	MHC I						MHC II											
**Patients**	**A**	**A**	**B**	**B**	**C**	**C**	**DRB1**	**DRB1**	**DRB345**	**DRB345**	**DQA**	**DQA**	**DQB**	**DQB**	**DPA**	**DPA**	**DPB**	**DPB**
MPM.001	**0301**	**3101**	**3901**	**4402**	**0702**	**0501**	**1401**	**1501**	**3*0202**	**5*0101**	**0104**	**0102**	**0503**	**0602**	**0103**	**0103**	**0301P**	**0401**
MPM.002	**0201**	**2402**	**1515**	**5201**	**0102**	**0303**	**0802**	**1406**		**3*0101**	**0401**	**0503**	**0402**	**0301**	**0103**	**0103**	**0402**	**0402**
MPM.003	**0101**	**3002**	**1801**	**4402**	**0501**	**0501**	**0301**	**0401**	**3*0202**	**4*0103**	**0501**	**0303**	**0201**	**0301**	**0103**	**0103**	**0202**	**0401**
MPM.004	**2601**	**6801**	**3503**	**3701**	**0401**	**0602**	**0801**	**1301**		**3*0202**	**0301**	**0103**	**0302**	**0603**	**0103**	**0103**	**0401**	**0402**
MPM.005	**0101**	**1101**	**1302**	**4402**	**1502**	**0704**	**0701**	**1101**	**4*0101**	**3*0202**	**0201**	**0505**	**0202**	**0301**	**0103**	**0201**	**0201**	**1701**
MPM.006	**0201**	**0301**	**0801**	**1302**	**0701**	**0602**	**0301**	**0701**	**3*0101**	**4*0101**	**0501**	**0201**	**0201**	**0202**	**0201**	**0201**	**1401**	**1401**
MPM.007	**0201**	**0201**	**4002**	**5001**	**0202**	**0602**	**0408**	**0701**	**4*0103**	**4*0101**	**0303**	**0201**	**0301**	**0202**	**0103**	**0103**	**0301P**	**0401**
MPM.008	**0101**	**0301**	**0801**	**3901**	**0701**	**1203**	**0301**	**1301**	**3*0101**	**3*0101**	**0501**	**0103**	**0201**	**0603**	**0103**	**0201**	**0201**	**1301P**
MPM.009	**0201**	**0301**	**0702**	**270502**	**0702**	**0102**	**0101**	**1501**	**--**	**5*0101**	**0101**	**0102**	**0501**	**0602**	**0103**	**0103**	**0401**	**0402**
MPM.010	**0101**	**0201**	**0801**	**4402**	**0701**	**0501**	**0101**	**0301**	**--**	**3*0101**	**0101**	**0501**	**0501**	**0201**	**0103**	**0201**	**0101**	**1601**
MPM.011	**0201**	**2402**	**0702**	**1801**	**0702**	**0701**	**1101**	**1501**	**3*0202**	**5*0101**	**0505**	**0102**	**0301**	**0602**	**0103**	**0103**	**0401**	**0401**

Supplementary Table 6. MHC I-binding neopeptides with high or intermediate binding affinity.

Table is provided in Other Supplementary Material as an Excel file
(Table S6 in Supplementary.Tables.xls).

Supplementary Table 7. MHC II-binding neopeptides with high or intermediate binding affinity.

Table is provided in Other Supplementary Material as an Excel file
(Table S7 in Supplementary.Tables.xls)

Supplementary Table 8. Differential protein and mRNA expression between two TiME subsets.

	Protein	Differential Protein Expression by Mass spectrometry					Differential mRNA Expression by mRNA Array				
		Parametric p-value	Permutation p-value	TiME-I	TiME-II	Fold-change	Parametric p-value	Permutation p-value	TiME-I	TiME-II	Fold-change
Up-regulation in TiME-I	ACADM	0.082	0.110	3.760	1.170	3.21	0.006	0.011	1.310	0.760	1.710
	AKAP1	0.048	0.067	0.012	0.000	116.64	0.004	0.009	1.160	0.940	1.230
	ALDH1A2	0.002	0.013	11.370	1.120	10.15	0.000	0.002	2.130	0.320	6.710
	ANXA2	0.093	0.097	717.860	316.17	2.27	0.017	0.022	1.420	0.480	2.960
	ANXA3	0.006	0.019	39.220	7.020	5.59	0.001	0.004	4.160	0.630	6.590
	ANXA8	0.006	0.013	19.770	0.006	3312.5	0.000	0.004	2.160	0.170	12.970
	ANXA8L2	0.028	0.013	52.940	0.350	150.02	0.000	0.002	2.730	0.420	6.460
	ANXA9	0.059	0.167	0.068	0.000	215.56	0.035	0.004	1.510	0.920	1.640
	APOA1BP	0.026	0.045	8.600	2.810	3.060	0.000	0.002	1.690	0.680	2.490
	APRT	0.043	0.078	18.290	10.790	1.700	0.001	0.006	1.280	0.740	1.740
	AQP1	0.076	0.024	18.320	0.390	47.350	0.028	0.035	1.500	0.640	2.340
	ARMC10	0.077	0.056	0.330	0.022	14.920	0.031	0.024	1.140	0.690	1.660
	ASH2L	0.087	0.100	0.039	0.003	13.030	0.001	0.002	1.550	0.770	2.000
	ATP1B1	0.081	0.074	1.740	0.028	62.510	0.048	0.039	1.190	0.660	1.800
	ATP5O	0.036	0.058	16.870	8.360	2.020	0.013	0.004	1.140	0.730	1.570
	B4GALT5	0.075	0.106	0.044	0.003	14.470	0.046	0.052	1.320	0.840	1.570
	BAG1	0.098	0.169	0.220	0.008	29.200	0.003	0.002	1.090	0.950	1.150
	BCAT2	0.094	0.050	1.160	0.056	20.610	0.009	0.017	1.330	0.860	1.550
	BSG	0.093	0.013	12.340	0.360	34.650	0.002	0.002	1.250	0.560	2.220
	C1GALT1	0.073	0.108	0.061	0.003	18.500	0.029	0.028	1.540	0.840	1.840
	C4A	0.001	0.024	13.560	0.001	16215	0.022	0.013	1.310	0.940	1.400
	CA9	0.025	0.048	0.050	0.000	154.14	0.005	0.011	2.380	0.660	3.600
	CADM3	0.002	0.035	0.005	0.000	53.120	0.022	0.006	1.510	0.930	1.620
	CALB2	0.016	0.024	112.490	1.670	67.440	0.000	0.002	3.310	0.240	14.000
	CD200	0.025	0.089	0.011	0.000	109.69	0.009	0.011	1.440	0.810	1.770
	CD38	0.071	0.110	0.019	0.000	66.750	0.039	0.030	1.410	0.880	1.600
	CDC42EP4	0.004	0.013	0.220	0.002	116.27	0.001	0.002	1.460	0.620	2.370
	CHEK2	0.075	0.305	0.001	0.000	8.920	0.006	0.004	1.100	0.850	1.290
	CLIC3	0.085	0.097	3.490	0.095	36.860	0.030	0.041	2.140	0.650	3.320
	COX5B	0.004	0.013	43.480	12.700	3.420	0.002	0.002	1.340	0.780	1.720
	CRIP1	0.033	0.013	98.260	0.570	172.68	0.000	0.002	2.170	0.420	5.200
	DHRS3	0.029	0.039	0.730	0.017	43.420	0.004	0.013	1.310	0.490	2.650
	EBAG9	0.050	0.130	0.020	0.001	38.350	0.006	0.002	1.250	0.830	1.520
	EPHX2	0.001	0.013	0.280	0.001	488.7	0.019	0.024	1.290	0.800	1.610
	EPS8L1	0.044	0.100	0.003	0.000	29.320	0.028	0.039	1.170	0.910	1.280
	ERLIN2	0.010	0.013	6.810	2.740	2.480	0.027	0.035	1.130	0.880	1.280
	EZR	0.005	0.019	88.630	28.690	3.090	0.037	0.045	1.250	0.760	1.640

FAM96A	0.029	0.078	0.270	0.002	114.97	0.035	0.041	1.100	0.750	1.480
FBXO2	0.010	0.013	4.250	0.010	434.99	0.002	0.004	1.730	0.550	3.170
GALK2	0.020	0.052	0.040	0.001	47.020	0.002	0.002	1.350	0.860	1.560
GCHFR	0.045	0.065	1.130	0.029	38.480	0.043	0.052	1.760	0.910	1.950
GFPT1	0.022	0.041	3.190	1.790	1.780	0.040	0.045	1.140	0.920	1.250
GNAI1	0.070	0.089	0.020	0.000	74.470	0.009	0.002	1.280	0.910	1.420
GNG12	0.087	0.037	11.650	6.010	1.940	0.015	0.011	1.160	0.800	1.460
GPD1	0.072	0.108	0.052	0.001	90.240	0.042	0.048	1.140	0.990	1.150
GPI	0.045	0.091	35.410	21.100	1.680	0.016	0.006	1.170	0.790	1.480
GSTZ1	0.004	0.032	0.400	0.001	353.46	0.013	0.013	1.090	0.930	1.180
HSD17B8	0.024	0.026	0.350	0.005	74.550	0.000	0.002	1.930	0.700	2.760
IFITM2	0.051	0.100	0.790	0.005	159.73	0.026	0.032	1.250	0.720	1.750
ISOC2	0.071	0.013	4.540	0.140	31.340	0.001	0.009	1.660	0.880	1.890
ISYNA1	0.060	0.097	3.040	0.290	10.480	0.002	0.006	1.650	0.740	2.210
KCNH5	0.075	0.167	0.010	0.000	101.40	0.020	0.026	1.070	0.980	1.100
KRT18	0.005	0.024	410.590	24.960	16.450	0.000	0.002	2.220	0.550	4.050
KRT19	0.000	0.013	906.610	25.790	35.160	0.000	0.002	2.590	0.260	10.070
KRT5	0.001	0.013	255.620	4.690	54.540	0.000	0.002	2.690	0.440	6.160
KRT8	0.002	0.013	1087.670	58.990	18.440	0.001	0.002	3.070	0.470	6.560
LAMA3	0.045	0.056	0.130	0.002	55.920	0.008	0.011	1.360	0.710	1.930
LDHA	0.045	0.069	248.380	110.29	2.250	0.047	0.054	1.390	0.850	1.640
LGALS3BP	0.031	0.035	9.690	3.610	2.690	0.008	0.013	1.550	0.610	2.550
LRRC1	0.000	0.013	0.160	0.000	476.96	0.009	0.013	1.960	0.510	3.830
LRRN4	0.046	0.069	0.690	0.009	73.370	0.002	0.009	2.670	0.930	2.870
MBP	0.011	0.013	0.096	0.002	51.510	0.005	0.006	1.540	0.640	2.400
MCFD2	0.099	0.147	6.600	3.220	2.050	0.025	0.022	1.120	0.840	1.340
MRPL41	0.076	0.134	0.600	0.012	50.750	0.038	0.039	1.260	0.910	1.380
MRPL46	0.047	0.056	0.170	0.007	25.060	0.020	0.015	1.240	0.970	1.280
MYO5B	0.087	0.130	0.004	0.000	13.250	0.006	0.013	1.800	0.910	1.970
NDUFA2	0.019	0.032	2.190	0.710	3.080	0.008	0.011	1.270	0.860	1.490
NDUFB3	0.075	0.082	1.740	0.025	68.510	0.022	0.013	1.150	0.840	1.380
NEBL	0.042	0.089	0.470	0.003	145.33	0.000	0.004	2.510	0.810	3.090
NEK7	0.079	0.069	1.380	0.049	28.300	0.014	0.009	1.270	0.870	1.470
NFATC2IP	0.078	0.208	0.001	0.000	13.650	0.019	0.024	1.220	0.870	1.400
PDHB	0.098	0.123	5.770	3.690	1.560	0.011	0.011	1.400	0.880	1.590
PGM1	0.074	0.169	30.080	12.440	2.420	0.002	0.002	2.450	0.760	3.200
PKP2	0.000	0.013	0.360	0.001	350.96	0.000	0.002	1.530	0.760	2.010
PPA1	0.079	0.113	15.500	7.880	1.970	0.027	0.030	1.390	0.700	1.980
PRDX3	0.072	0.017	22.440	8.350	2.690	0.045	0.050	1.120	0.670	1.680
PRDX5	0.021	0.061	68.060	30.260	2.250	0.014	0.024	1.740	0.920	1.890
PTGIS	0.031	0.069	18.710	2.190	8.560	0.017	0.030	1.660	0.530	3.120

	PYCR2	0.057	0.061	0.580	0.011	52.140	0.017	0.006	1.100	0.870	1.270
	RAB7B	0.082	0.273	0.002	0.000	22.200	0.039	0.050	1.460	0.640	2.270
	RARRES1	0.021	0.058	0.016	0.000	159.89	0.000	0.002	5.530	0.410	13.460
	RBM47	0.052	0.080	0.048	0.002	22.720	0.033	0.039	1.480	0.780	1.890
	RDH10	0.000	0.013	0.420	0.000	4208.8	0.005	0.004	2.320	0.600	3.850
	RNF39	0.074	0.229	0.009	0.000	89.800	0.005	0.006	1.050	0.950	1.110
	RTN3	0.075	0.089	2.390	0.690	3.480	0.007	0.009	1.290	0.770	1.660
	SCP2	0.047	0.084	10.740	6.140	1.750	0.028	0.035	1.180	0.920	1.280
	SEC11C	0.002	0.013	1.010	0.001	819.15	0.003	0.002	1.260	0.660	1.910
	SELENBP1	0.040	0.097	17.930	2.520	7.110	0.029	0.032	2.130	0.840	2.530
	SIRT5	0.053	0.093	0.075	0.002	48.730	0.001	0.002	1.290	0.850	1.520
	SLC16A1	0.012	0.030	0.990	0.005	188.14	0.002	0.009	1.130	0.960	1.180
	SLC19A3	0.079	0.242	0.002	0.000	18.110	0.046	0.037	1.300	0.940	1.390
	SLC9A3R1	0.002	0.024	16.020	3.520	4.550	0.011	0.015	1.540	0.570	2.700
	SMPDL3B	0.008	0.067	0.061	0.000	318.22	0.036	0.045	1.090	0.910	1.200
	SORBS2	0.058	0.091	1.060	0.028	38.260	0.000	0.002	2.270	0.450	5.030
	SPTAN1	0.041	0.067	18.080	7.990	2.260	0.026	0.032	1.210	0.770	1.560
	STAT3	0.012	0.019	5.230	1.860	2.810	0.032	0.015	1.280	0.840	1.520
	SUCLG1	0.013	0.041	6.500	3.870	1.680	0.007	0.013	1.210	0.890	1.360
	SULT1A1	0.064	0.013	14.930	0.300	50.050	0.002	0.002	1.640	0.670	2.460
	SULT1A3	0.001	0.013	3.960	0.350	11.350	0.000	0.002	2.150	0.700	3.080
	SULT1A4	0.001	0.013	3.960	0.350	11.350	0.000	0.002	1.940	0.570	3.390
	TBL2	0.079	0.087	1.170	0.590	1.990	0.028	0.035	1.130	0.890	1.270
	TM4SF1	0.000	0.013	1.900	0.001	2905.8	0.010	0.006	1.290	0.460	2.790
	TST	0.027	0.041	4.830	1.990	2.420	0.009	0.004	1.510	0.730	2.070
	TSTD1	0.045	0.013	3.640	0.028	130.07	0.000	0.002	1.720	0.720	2.380
	TUFM	0.012	0.019	17.620	8.020	2.200	0.041	0.045	1.100	0.940	1.180
	UPK1B	0.019	0.165	0.053	0.000	530.72	0.000	0.002	3.760	0.590	6.390
	UQCRH	0.051	0.069	0.099	0.004	25.920	0.030	0.030	1.120	0.800	1.400
	WNT2B	0.092	0.199	0.004	0.000	42.530	0.002	0.006	1.340	0.570	2.370
Down-regulation in TiME-I	ARFGAP1	0.064	0.071	0.610	1.620	0.380	0.001	0.006	0.810	1.290	0.630
	CALU	0.099	0.128	44.280	84.550	0.520	0.039	0.041	0.710	1.490	0.480
	CLASP1	0.054	0.061	0.042	0.240	0.180	0.047	0.006	0.830	1.500	0.560
	DBN1	0.046	0.089	2.890	12.230	0.240	0.048	0.050	0.850	1.750	0.490
	EIF4G3	0.025	0.026	0.140	0.840	0.170	0.017	0.024	0.860	1.130	0.770
	ENAH	0.046	0.076	0.330	1.290	0.250	0.041	0.039	0.920	1.470	0.630
	FCHO2	0.077	0.048	0.016	0.460	0.035	0.005	0.011	0.820	1.220	0.680
	GDI2	0.078	0.054	22.730	36.610	0.620	0.013	0.006	0.740	1.070	0.690
	IPO13	0.096	0.149	0.000	0.003	0.063	0.031	0.017	0.840	1.210	0.690
	ITGA11	0.020	0.045	0.000	0.015	0.010	0.038	0.048	0.820	1.620	0.510
	LEPRE1	0.005	0.015	0.440	2.090	0.210	0.012	0.015	0.780	1.280	0.610

	LEPREL2	0.089	0.100	0.009	0.360	0.024	0.024	0.037	0.710	1.360	0.520
	LGALS1	0.033	0.045	131.630	239.78	0.550	0.042	0.050	0.680	1.140	0.600
	LPP	0.054	0.071	4.620	10.500	0.440	0.015	0.024	0.920	1.300	0.700
	MARCKS	0.035	0.045	12.300	23.840	0.520	0.009	0.009	0.860	1.330	0.650
	MGA	0.064	0.121	0.000	0.001	0.090	0.010	0.006	0.880	1.240	0.710
	MICAL1	0.100	0.104	0.120	0.950	0.120	0.047	0.048	0.800	1.270	0.630
	MYH9	0.010	0.032	67.650	138.96	0.490	0.006	0.006	0.810	1.450	0.560
	MYO1B	0.049	0.030	0.310	0.940	0.330	0.004	0.004	0.460	1.290	0.360
	NBAS	0.031	0.041	0.015	0.078	0.190	0.000	0.002	0.930	1.120	0.830
	NOP14	0.020	0.043	0.004	0.240	0.018	0.013	0.013	0.900	1.090	0.830
	NUP54	0.032	0.039	0.280	0.590	0.470	0.034	0.041	0.870	1.120	0.780
	RCN1	0.065	0.119	19.790	31.690	0.620	0.005	0.009	0.850	1.210	0.710
	RFTN1	0.086	0.091	0.340	0.940	0.360	0.002	0.004	0.790	1.350	0.590
	TCERG1	0.096	0.123	0.610	1.080	0.560	0.015	0.006	0.900	1.060	0.840
	UBE3A	0.069	0.076	0.072	0.280	0.260	0.004	0.006	0.810	1.170	0.690
	VCL	0.008	0.026	12.650	30.490	0.410	0.000	0.002	0.650	1.610	0.400
	YARS	0.012	0.022	3.680	7.500	0.490	0.046	0.054	0.920	1.210	0.760
	ZW10	0.072	0.089	0.030	0.140	0.220	0.049	0.054	0.840	1.100	0.760

Supplementary Table 9. Univariable and multivariable Cox regression analysis of TiME signature and overall survival in the combined validation set (n=330).

**Variables**	**Univariable**		**Multivariable**	
	Hazard ratio (95% CI)	*P* value	Hazard ratio (95% CI)	*P* value
Sex (Male vs. Female)	1.16 (0.85-1.57)	0.351		
Age (≥65 y vs.<65 y)	1.82 (1.41-2.34)	<0.001	1.74 (1.32-2.30)	<0.001
Asbestos Exposure History (Yes *vs.* No)	1.49 (1.13-1.96)	0.005	1.19 (0.89-1.59)	0.239
pT stage			overall	0.186
1	1	-	1	
2	1.40 (0.85-2.23)	0.186	1.45 (0.86-2.44)	0.165
3	1.72 (1.06-2.79)	0.027	1.53 (0.92-2.55)	0.099
4	2.22 (1.30-3.79)	0.003	1.87 (1.06-3.28)	0.030
pN stage				
0	1	-		
1	1.05 (0.72-1.54)	0.787		
2	1.18 (0.87-1.59)	0.282		
3	1.20 (0.44-3.26)	0.722		
Histology (Non-epithelioid vs. Epithelioid)	1.90 (1.47-2.47)	<0.001	1.50 (1.10-2.04)	0.010
TiME signature (Bad- vs. Good-TiME)	1.61 (1.26-2.05)	<0.001	1.74 (1.32-2.30)	0.017

CI, confidence interval.

References

58. Sugarbaker DJ, Richards WG, Bueno R. Extrapleural pneumonectomy in the treatment of epithelioid malignant pleural mesothelioma: novel prognostic implications of combined N1 and N2 nodal involvement based on experience in 529 patients. Ann Surg **2014**;260(4):577-580.
59. Rice D, *et al.* Recommendations for uniform definitions of surgical techniques for malignant pleural mesothelioma: a consensus report of the international association for the study of lung cancer international staging committee and the international mesothelioma interest group. J Thorac Oncol **2011**;6(8):1304-1312.
60. Jang H-J, *et al.* Integrated genomic analysis of recurrence-associated small non-coding RNAs in oesophageal cancer. Gut **2017**;66(2):215-225.
61. Chew V, *et al.* Delineation of an immunosuppressive gradient in hepatocellular carcinoma using high-dimensional proteomic and transcriptomic analyses. Proc Natl Acad Sci U S A **2017**;114:E5900-E5909.
62. Levine JH, *et al.* Data-driven phenotypic dissection of AML reveals progenitor-like cells that correlate with prognosis. Cell **2015**;162(1):184-197.
63. Zunder ER, Finck R, Behbehani GK, El-ad DA, Krishnaswamy S, Gonzalez VD, *et al.* Palladium-based mass tag cell barcoding with a doublet-filtering scheme and single-cell deconvolution algorithm. Nat Protoc **2015**;10(2):316-333.
64. Finck R, *et al.* Normalization of mass cytometry data with bead standards. Cytometry Part A **2013**;83(5):483-494.
65. Bendall SC, *et al.* Single-cell mass cytometry of differential immune and drug responses across a human hematopoietic continuum. Science **2011**;332(6030):687-696.
66. Qiu P, *et al.* Extracting a cellular hierarchy from high-dimensional cytometry data with SPADE. Nat Biotechnol **2011**;29(10):886-891.
67. Maecker HT, McCoy JP, Nussenblatt R. Standardizing immunophenotyping for the human immunology project. Nat Rev Immunol **2012**;12:191-200.
68. Mason GM, *et al.* Phenotypic complexity of the human regulatory T cell compartment revealed by mass cytometry. J Immunol **2015**;195(5):2030-2037.
69. Newell EW, Sigal N, Bendall SC, Nolan GP, Davis MM. Cytometry by time-of-flight shows combinatorial cytokine expression and virus-specific cell niches within a continuum of CD8+ T cell phenotypes. Immunity **2012**;36(1):142-152.
70. Sen N, *et al.* Single-cell mass cytometry analysis of human tonsil T cell remodeling by varicella zoster virus. Cell Rep **2014**;8(2):633-645.
71. Huang AC, *et al.* T-cell invigoration to tumour burden ratio associated with anti-PD-1 response. Nature **2017**;545(7652):60-65.
72. Corneau A, *et al.* Comprehensive mass cytometry analysis of cell cycle, activation and co-inhibitory receptors expression in CD4 T cells from healthy and HIV-infected individuals. Cytometry B Clin Cytom **2016**;92:236-237.
73. Murray PJ, Wynn TA. Protective and pathogenic functions of macrophage subsets. Nat Rev Immunol **2011**;11:723-737.
74. Moore AJ, Anderson MK. Dendritic cell development: a choose-your-own-adventure story. Adv Hematol **2013**;2013.
75. Giesen C, *et al.* Highly multiplexed imaging of tumor tissues with subcellular resolution by mass cytometry. Nat Methods **2014**;11(4):417-422.
76. Husain AN, *et al.* Guidelines for pathologic diagnosis of malignant mesothelioma: 2012 update of the consensus statement from the International Mesothelioma Interest Group. Arch Pathol Lab Med **2012**;137(5):647-667.

77. Munz M, Baeuerle PA, Gires O. The emerging role of EpCAM in cancer and stem cell signaling. Cancer Res **2009**;69(14):5627-9.
78. Dalerba P, *et al.* Phenotypic characterization of human colorectal cancer stem cells. Proc Natl Acad Sci U S A **2007**;104:10158-10163.
79. Ghani FI, *et al.* Identification of cancer stem cell markers in human malignant mesothelioma cells. Biochem Biophys Res Commun **2011**;404(2):735-742.
80. Yamazaki H, Naito M, Ghani FI, Dang NH, Iwata S, Morimoto C. Characterization of cancer stem cell properties of CD24 and CD26-positive human malignant mesothelioma cells. Biochem Biophys Res Commun **2012**;419(3):529-536.
81. Varghese S, Whipple R, Martin SS, Alexander HR. Multipotent cancer stem cells derived from human malignant peritoneal mesothelioma promote tumorigenesis. PLoS One **2012**;7(12):e52825.
82. Gedye CA, *et al.* Cell surface profiling using high-throughput flow cytometry: a platform for biomarker discovery and analysis of cellular heterogeneity. PLoS One **2014**;9(8):e105602.
83. Gunaydin G, Kesikli SA, Guc D. Cancer associated fibroblasts have phenotypic and functional characteristics similar to the fibrocytes that represent a novel MDSC subset. Oncoimmunology **2015**;4(9):e1034918.
84. Lua I, Li Y, Pappoe LS, Asahina K. Myofibroblastic conversion and regeneration of mesothelial cells in peritoneal and liver fibrosis. Am J Pathol **2015**;185:3258-3273.
85. Lua I, *et al.* Characterization of hepatic stellate cells, portal fibroblasts, and mesothelial cells in normal and fibrotic livers. J Hepatol **2016**;64(5):1137-1146.
86. Li Y, Wang J, Asahina K. Mesothelial cells give rise to hepatic stellate cells and myofibroblasts via mesothelial–mesenchymal transition in liver injury. Proc Natl Acad Sci U S A **2013**;110:2324-2329.
87. Lee H-S, *et al.* Genomic Analysis of Thymic Epithelial Tumors Identifies Novel Subtypes Associated with Distinct Clinical Features. Clin Cancer Res **2017**;23:4855-4864.
88. Cheadle C, Vawter MP, Freed WJ, Becker KG. Analysis of microarray data using Z score transformation. The Journal of molecular diagnostics **2003**;5(2):73-81.
89. Jung SY, *et al.* An anatomically resolved mouse brain proteome reveals Parkinson disease-relevant pathways. Mol Cell Proteomics **2017**;16(4):581-593.
83. 90. Ramaswamy S, *et al.* Multiclass cancer diagnosis using tumor gene expression signatures. Proc Natl Acad Sci U S A **2001**;98(26):15149-15154.
91. Alexandrov LB, *et al.* Signatures of mutational processes in human cancer. Nature **2013**;500(7463):415-421.
92. Roh W, *et al.* Integrated molecular analysis of tumor biopsies on sequential CTLA-4 and PD-1 blockade reveals markers of response and resistance. Sci Transl Med **2017**;9(379):eaah3560.
93. Bastian BC, LeBoit PE, Hamm H, Bröcker E-B, Pinkel D. Chromosomal gains and losses in primary cutaneous melanomas detected by comparative genomic hybridization. Cancer Res **1998**;58(10):2170-2175.
94. Shain AH, *et al.* The genetic evolution of melanoma from precursor lesions. N Engl J Med **2015**;373(20):1926-1936.
95. Li B, *et al.* Landscape of tumor-infiltrating T cell repertoire of human cancers. Nat Genet **2016**;48(7):725-732.
96. McNeel DG. TCR diversity–a universal cancer immunotherapy biomarker? J Immunother Cancer **2016**;4(1):69.

97. Mermel CH, Schumacher SE, Hill B, Meyerson ML, Beroukhim R, Getz G. GISTIC2.0 facilitates sensitive and confident localization of the targets of focal somatic copy-number alteration in human cancers. Genome Biol **2011**;12(4):R41.
